# Chemical Profiling and UPLC-qTOF-MS/MS-Based Metabolomics of Three Different Parts of *Edgeworthia chrysantha* and Identification of Glucose Uptake-Enhancing Compounds

**DOI:** 10.3390/nu17162684

**Published:** 2025-08-19

**Authors:** Jin-Pyo An, Sohee Han, Van-Hieu Mai, Jorge-Eduardo Ponce-Zea, Gi Hyeon Seong, Thi-Kim-Quy Ha, Won Keun Oh

**Affiliations:** 1 Research Institute of Pharmaceutical Sciences, College of Pharmacy, Seoul National University, Seoul 151-742, Republic of Korea; ntopjp77@snu.ac.kr (J.-P.A.); sohee.hn@snu.ac.kr (S.H.); maihieu@snu.ac.kr (V.-H.M.); jepz210689@snu.ac.kr (J.-E.P.-Z.); ghseong00@snu.ac.kr (G.H.S.); htkquy@ctu.edu.vn (T.-K.-Q.H.); 2 College of Natural Sciences, Can Tho University, Campus II, Can Tho 90000, Vietnam

**Keywords:** *Edgeworthia chrysantha*, metabolomics, glucose uptake, anti-diabetic, diabetes, Thymelaeaceae

## Abstract

Background/Objectives: *Edgeworthia chrysantha* is rich in coumarin and flavonoid dimers, which may exhibit diverse pharmacological activities. However, to date, no metabolomics studies have been conducted and its bioactive constituents related to glucose metabolism remain uncharacterized. This study aimed to conduct a comprehensive chemical analysis combined with bioactivity assays to evaluate its efficacy in promoting glucose uptake. Methods: Chemical profiling of three parts (leaf, stem, and root) of *E. chrysantha* was performed using UPLC-Q-TOF-MS/MS spectrometry, followed by metabolomics analysis. Based on the chemical profiles and glucose uptake activity, compounds were isolated from the root. Their structures were elucidated using spectroscopic techniques, including UV, NMR, and mass spectrometry. The glucose uptake activity of the isolated compounds was assessed using a 2-NBDG assay. Results: Metabolic analysis revealed distinct chemical compositions among the plant parts. Dimeric coumarins and biflavonoids were abundant in the root, whereas flavonoid monomers were predominant in the leaf. Bioactivity-guided isolation yielded nine compounds (**1**–**9**), among which compound **1**, a newly identified coumarin glycoside, exhibited significant glucose uptake-enhancing activity. Molecular docking analysis further suggested that compound **1** activates AMPK through an allosteric site, thereby promoting glucose uptake. Conclusions: These findings provide a comprehensive chemical and metabolomic characterization of *E. chrysantha* and highlight its potential as a functional food ingredient for glucose-lowering effects.

## 1. Introduction

Diabetes remains a major global health challenge, with an estimated 589 million adults affected in 2024, nearly half of whom remain undiagnosed. Additionally, approximately 1.1 billion adults worldwide are living with impaired glucose tolerance or impaired fasting glucose, placing them at elevated risk of developing type 2 diabetes mellitus (T2DM) [1]. Therefore, enhancing glucose uptake in insulin-responsive tissues offers a promising therapeutic approach for lowering blood glucose levels and developing novel antidiabetic agents.

Recent studies have shown that coumarins possess notable anti-diabetic properties, in addition to their anti-inflammatory and antioxidant activities. For instance, esculin, a glycosylated coumarin, was found to enhance glucose uptake in normal C2C12 myotubes and improve insulin resistance in dexamethasone-treated cells [2]. Similarly, osthole, a non-glycosylated coumarin, promoted glucose uptake by activating AMP-activated protein kinase (AMPK) in skeletal muscle cells [3]. Beyond enhancing cellular glucose uptake, coumarins also modulate carbohydrate digestion at the intestinal level. They have been found to inhibit the activity of α-glucosidase and α-amylase, which are essential enzymes involved in breaking down disaccharides into glucose during carbohydrate metabolism [4]. Coumarins exist not only as monomers but also as dimers and trimers, exhibiting considerable structural diversity. These oligomeric forms, typically linked through carbon–carbon (C–C) or ether (C–O–C) bonds, may display stronger or broader biological activities than their simpler counterparts, highlighting their promising pharmacological potential [5]. In addition to coumarins, biflavonoids represent a major class of polyphenols abundant in the *Edgeworthia* genus and other Thymelaeaceae species. These dimeric compounds, formed through oxidative coupling of flavonoid monomers, exhibit remarkable structural diversity [6]. Biflavonoids have been reported to exhibit a wide range of pharmacological properties, including anti-diabetic effects [7,8]. *E. chrysantha* Lindl., also known as *E. papyrifera* S. et Z., is a deciduous, suckering shrub of the family Thymelaeaceae, native to the forested areas and bushy slopes of Southwest China. It is widely cultivated across China, Japan, and Korea due to its applications in traditional medicine, papermaking, and ornamental horticulture [9]. Despite its phytochemical richness, *E. chrysantha* remains largely unexplored in terms of metabolomic analysis and few studies have identified its anti-diabetic constituents.

To address this gap, we conducted a study consisting of three main components: compound isolation, metabolomics analysis, and bioassay evaluation. In this study, nine compounds (**1**–**9**) were isolated through bioactivity-guided fractionation, including five coumarin glycosides, one coumarin aglycone, and three flavonoid dimers. Notably, compound **1** was identified as a novel coumarin glycoside, reported here for the first time from a natural source. Subsequently, UPLC-qTOF-MS/MS-based chemical profiling of the leaf, stem, and root of *E. chrysantha* was performed, and the resulting metabolomics analysis revealed distinct chemical characteristics among the three plant parts. Finally, bioassay results demonstrated that compound **1** significantly enhanced glucose uptake. Molecular docking analysis further revealed that this effect is mediated through AMPK activation, contributing to its glucose-lowering activity.

## 2. Materials and Methods

### 2.1. General Experimental Procedures

Analytical-grade solvents were purchased from Sigma-Aldrich (St. Louis, MO, USA), while extraction-grade solvents were supplied by Daejung Chemicals & Metals Co. (Siheung, Republic of Korea). A JASCO P-2000 polarimeter was used to determine optical rotation, and infrared (IR) spectra were recorded on a Nicolet 6700 FT-IR spectrometer (Thermo Scientific, Walthan, MA, USA). ^1^H and ^13^C NMR spectra were acquired using JNM-ECA-600 (JEOL, Tokyo, Japan) and Bruker Avance-500 (Bruker, Billerica, MA, USA) instruments at Seoul National University (Seoul, Republic of Korea). For column separation, reversed-phase silica gel (ODS-A, S-150 μm; YMC, Kyoto, Japan), Diaion HP-20 (Mitsubishi Chemical, Tokyo, Japan), and Sephadex LH-20 (Amersham Pharmacia Biotech, Uppsala, Sweden) were used. Medium-pressure liquid chromatography (MPLC) was conducted on a Reveleris system (Grace, IL, USA) equipped with a C18 flash cartridge (120 g). Semi-preparative HPLC was carried out using a Gilson 321 pump and UV/VIS-151 detector (Gilson Inc., Middleton, WI, USA), along with a YMC-Triart C18 column (250 × 10 mm ID, 5 μm, YMC).

### 2.2. Plant Material

Whole-plant specimens of *Edgeworthia chrysantha* Lindl. were collected in March from five-year-old trees cultivated at the Medicinal Plant Garden, College of Pharmacy, Seoul National University, Goyang-si, Gyeonggi-do, Republic of Korea (37°42′40″ N, 126°49′07″ E). The botanical identity of the specimen was confirmed by Sang-il Han from the same institution. A voucher specimen (SNU2024-12) has been deposited in the Laboratory of Pharmacognosy, College of Pharmacy, Seoul National University, Seoul, Republic of Korea.

### 2.3. Isolation of Chemical Constituents from the Root Part of Edgeworthia chrysantha

Three consecutive ultrasonic-assisted extractions (90 min each) were performed on 380 g of air-dried, finely powdered *E. chrysantha* roots using 2 L of 90% EtOH per cycle (Figure S1). Extractions were conducted with a Powersonic 420 ultrasonic cleaner (Hwashin Tech, Seoul, Republic of Korea) operating at a frequency of 40 kHz at room temperature. The combined extracts were filtered and evaporated under reduced pressure to yield a crude residue of 28 g. This residue was then successively partitioned with n-hexane (3 × 1 L), ethyl acetate (EtOAc; 3 × 1 L), n-butanol (n-BuOH; 3 × 1 L), and water. Each solvent layer was concentrated under vacuum. The EtOAc fraction (5.0 g) was subsequently subjected to further separation by medium-pressure liquid chromatography (MPLC; Biotage, Uppsala, Sweden) using an ISU-C18 column (120 g) at a flow rate of 20 mL/min, with UV detection at 254 nm and 360 nm. Elution was performed using a MeOH/H_2_O gradient (0.1% formic acid), increasing from 10% to 80% MeOH.

Twelve fractions (Fractions 1–12) were obtained based on their TLC profiles. Fraction 9 (648.6 mg) was further purified by Sephadex LH-20 column chromatography using 100% MeOH, yielding eight subfractions (9.1–9.8). Subfraction 9.3 (286.5 mg) was subjected to HPLC on a YMC-Triart Phenyl column with an isocratic mobile phase of MeCN/H_2_O (28:72) at a flow rate of 4.0 mL/min for 55 min, affording compounds **1** (6.8 mg), **5** (5.1 mg), and **6** (2.3 mg). Similarly, fraction 8 (854.8 mg) was separated by LH-20 chromatography with 100% MeOH into five subfractions (8.1–8.5). Subfraction 8.3 (109.2 mg) was further purified by HPLC on a YMC-Triart Phenyl column (MeOH/H_2_O, 40/60; 4.0 mL/min; 60 min), resulting in the isolation of a dicoumarinyl ether glucoside (compound **2**, 5.3 mg). Subfraction 8.4 (331.7 mg) was purified by HPLC using a YMC-Triart C18 column (MeCN/H_2_O, 21/79; 4.0 mL/min; 50 min), affording biflavonoids **7** (8.2 mg), **8** (6.7 mg), and **9** (5.9 mg). Compound **3** (22.0 mg) was isolated from fraction 12 (370.0 mg) following separation via LH-20 chromatography under 100% MeOH. Fraction 7 (483.1 mg) was also purified using LH-20 under the same isocratic conditions. Subfraction 7.2 (88.6 mg) was further refined by HPLC (MeOH/H_2_O, 53/47), yielding compound **4** (4.2 mg). Finally, fraction 10 (104.2 mg) was chromatographed using LH-20 with 100% MeOH, producing nine subfractions (10.1–10.9). Subfraction 10.4 (44.2 mg) was further purified by HPLC on a YMC-Triart Phenyl column (MeCN/H_2_O, 25/60; 4.0 mL/min; 60 min), from which compound **5** (5.1 mg) was re-isolated. ^1^H and ^13^C NMR spectra and ECD spectra were shown in Figures S2–S19.

### 2.4. Sugar Analysis

To determine the sugar moiety of compound **1** (2 mg), acid hydrolysis was performed by treating 2 mg of compound **1** with 1 N HCl (200 µL) and heating the mixture in a water bath at 90 °C for 1.5 h. Upon completion, the reaction was neutralized with a saturated sodium carbonate solution. The progress of hydrolysis was monitored by TLC, which confirmed the disappearance of the original compound spot. Following hydrolysis, derivatization was carried out to identify the sugar. The hydrolysate, along with standard d-(+)-glucose (1 mg) and L-(–)-glucose (1 mg), was individually reacted with L-cysteine methyl ester hydrochloride (2 mg) in pyridine (200 µL). The mixtures were heated in an oven at 60 °C for 1 h. Subsequently, o-tolyl isothiocyanate (200 µL) was added to each reaction mixture, followed by an additional incubation at 60 °C for 1 h, according to the method of Tanaka et al. [10]. The resulting thiocarbamoyl thiazolidine derivatives were analyzed by analytical HPLC using a YMC-Triart C18 column (250 × 4.6 mm i.d.; YMC) at 35 °C. Separation was achieved under isocratic conditions using 25% MeCN in aqueous 0.1% formic acid as the mobile phase, at a flow rate of 1.0 mL/min for 30 min. The derivative of the hydrolyzed sample eluted at 21.1 min, matching the retention time of the d-(+)-glucose derivative, thereby confirming the sugar component as d-glucose.

### 2.5. Assessment of In Vitro Cytotoxic Effects in 3T3-L1 Adipocytes

The MTT assay was employed to evaluate the cytotoxicity of the isolated compounds in 3T3-L1 adipocytes. Cells were cultured in DMEM containing 10% FBS and seeded into 96-well plates, followed by incubation for 24 h at 37 °C under a 5% CO_2_ environment. Following incubation, cells were exposed to compounds **1**–**9** in serum-free medium and incubated for a further 24 h. Subsequently, 20 µL of MTT solution (2 mg/mL) was added to each well, and the plates were incubated in the dark for 4 h. After removing the medium, the resulting formazan crystals were dissolved in DMSO. The absorbance was measured at 550 nm using a microplate reader to assess cell viability following treatment with the test compounds.

### 2.6. Assessment of 2-NBDG Uptake in 3T3-L1 Adipocytes

The fluorescent D-glucose analog, 2-NBDG, was used to quantify glucose uptake in 3T3-L1 adipocytes. Cells were cultured in glucose-free medium with 10% FBS in 96-well plates and incubated for 24 h at 37 °C under 5% CO_2_ atmosphere. After treatment with compounds **1**–**9** or insulin (positive control) in the presence of 2-NBDG, cells were incubated for 1 hr, washed with cold PBS, and fluorescence was detected at 450/535 nm using a microplate reader.

### 2.7. Metabolomics Analysis Using UPLC qTOF MS/MS Spectrometry

Each plant part (stem, leaf, and root) of *E. chrysantha* (100 mg) was extracted with 1.0 mL of MeOH/H_2_O (70/30, *v*/*v*) by sonication for 3 h. Prior to analysis, 20 μL of internal standards, genistein-*d*_4_ and ^13^C-fructose (10 μg/mL), were added to each extract. The mixtures were centrifuged for 5 min, and the resulting supernatants were filtered through a 0.2 µm PTFE membrane filter before LC analysis. Samples were analyzed using a Xevo G3 QTOF mass spectrometer (Waters, Milford, MA, USA) equipped with an ACQUITY™ BEH C18 column (2.1 × 150 mm, 1.7 µm particle size). A binary solvent system consisting of 0.1% formic acid in H_2_O (solvent A) and 0.1% formic acid in MeCN (solvent B) was used. The gradient program was as follows: 0–1 min 10% B, 1–5 min 10–40% B, 5–20 min 40–98% B, 20–27 min 98% B, followed by a 3 min re-equilibration at the initial conditions. The flow rate was set to 0.4 mL/min, with an injection volume of 1 μL. Electrospray ionization (ESI) settings were: capillary voltage 2.5 kV (positive and negative modes); cone voltage 40 V; source temperature 120 °C; desolvation gas temperature, 350 °C; cone gas flow, 50 L/h; and desolvation gas flow, 800 L/h. During data acquisition, centroiding was applied using an independent reference lock-mass ion via the LockSpray™ interface (Waters) to ensure high mass accuracy and precision. Pooled quality control (QC) samples were injected every nine samples to monitor analytical consistency and detect potential instrumental drift.

### 2.8. Data Processing and Statistical Analysis

Raw LC–MS data files were converted into mzML format using MSConvert from the ProteoWizard toolkit (Version 3.0, Palo Alto, CA, US). Feature-based molecular networking (FBMN) analysis was conducted on the GNPS online platform (https://gnps.ucsd.edu), following spectral preprocessing with MZmine 4. Multivariate statistical analyses, including principal component analysis (PCA) and partial least squares-discriminant analysis (PLS-DA), were performed using ion intensity data exported in CSV format. Prior to analysis, compounds with missing values were excluded, and only variables with an RSD < 30% were retained. Data normalization and scaling were performed using MetaboAnalyst. These analyses were conducted on the MetaboAnalyst 5.0 web-based platform (https://www.metaboanalyst.ca), as described by Ewald et al. [11].

### 2.9. Molecular Docking Analysis

Molecular docking was performed using BIOVIA Discovery Studio 4.0 (Accelrys, San Diego, CA, USA). The crystal structure of AMP-activated protein kinase (AMPK) (PDB ID: 5ISO) was retrieved from the RCSB Protein Data Bank (http://www.rcsb.org). Before docking, heteroatoms, including water molecules, ions, and the co-crystallized reference ligand (compound 991), were removed. The allosteric binding site (ADaM site), located at the interface between the kinase domain of the α-subunit and the CBM domain of the β-subunit, was selected based on previous literature [12]. Flexible docking was performed using the CHARMm-based molecular dynamics protocol (CDOCKER). Protein–ligand interactions were optimized and docking scores were evaluated based on CDOCKER interaction energy values. Binding affinity was further assessed by analyzing key interactions, such as conventional hydrogen bonding, π-alkyl interactions, and van der Waals forces.

## 3. Results

### 3.1. Isolation of Bioactive Compounds and Structure Determination of New Compound 1 from the Roots of Edgeworthia chrysantha

**Figure 1:**
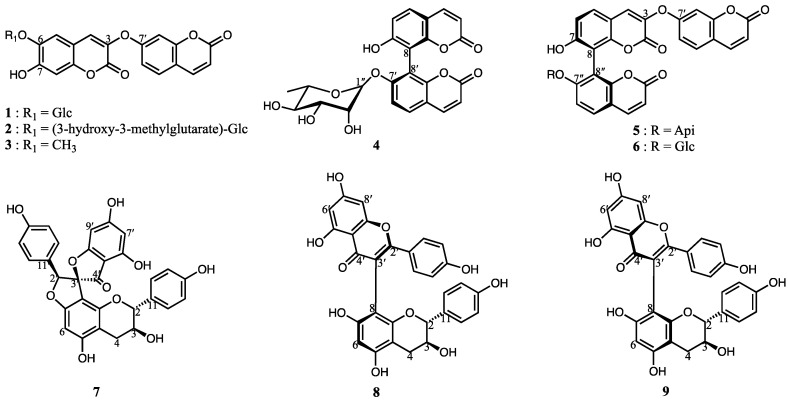
Nine secondary metabolites (**1**–**9**) were isolated and identified from the roots of *Edgeworthia chrysantha*, including various coumarin glycosides, aglycones, and biflavonoid derivatives.

Bioactivity-guided fractionation of *E. chrysantha* roots yielded nine compounds (**1**–**9**). Compound **1** is a newly identified natural product, while the remaining eight compounds were characterized by comparison with previously reported data. These known compounds include daphneretusin A (**2**) [13], daphnoretin (**3**) [14], edgeworoside C (**4**) [15], edgeworoside B (**5**) [15], 8,8′-bi-2H-1-benzopyran-2,2′-dione, 7′-(β-D-glucopyranosyloxy)-7-hydroxyl-3-(2-oxo-2H-1-benzopyran-7-yl)oxy (**6**) [16], daphnodorin I (**7**) [17], wikstrol A (**8**) [18], and wikstrol B (**9**) [18] (Figure 1). Since compounds **4**–**9** exhibit axial chirality, their absolute configurations were determined by electronic circular dichroism (ECD) analysis and were further confirmed through NMR spectroscopy and comparison with the literature.

Compound **1** was isolated as a colorless amorphous powder. Its molecular formula was determined to be C_30_H_28_O_16_ based on the negative-mode HRESIMS, which showed a peak at *m*/*z* 643.1299 [M − H]^−^ (calcd. for C_30_H_27_O_16_, 643.1299). The UV spectrum displayed absorption maxima at 338 nm (log ε 3.57) and 263 nm (log ε 3.17), while the IR spectrum exhibited bands corresponding to hydroxyl groups (3340 cm−^1^) and an α,β-unsaturated lactone (1730 cm^−1^), suggesting that the compound is a coumarin derivative. The ^1^H NMR spectrum revealed an ABX system at δ_H_ 7.70 (H-5′), 7.11 (H-6′), and 7.19 (H-8′), with corresponding coupling constants of *J* = 8.7 Hz (H-5′), *J* = 8.7, 2.4 Hz (H-6′), and *J* = 2.5 Hz (H-8′). In addition, *cis*-olefinic protons were observed at δ_H_ 8.03 (H-4′) and 6.37 (H-3′), with coupling constants of 9.6 and 9.4 Hz, respectively. Together with a lactone carbonyl resonance at δ_C_ 160.0 (C-2′) in the ^13^C NMR spectrum, these signals collectively indicated the presence of a benzopyran moiety. Furthermore, three aromatic protons appeared as singlets at δ_H_ 7.88 (H-4), 7.34 (H-6), and 6.91 (H-8). In combination with a second lactone carbonyl signal at δ_C_ 156.9 (C-2), these features indicated a 3,6,7-trisubstituted coumarin unit. The NMR data of compound **1** closely resembled those of edgeworthin, a compound previously isolated from *Edgeworthia gardneri*, supporting the presence of a dimeric coumarin moiety [19]. A doublet at δ_H_ 4.83 (1H, d, *J* = 7.4 Hz, H-1″) corresponded to the anomeric proton of a glucose residue, with the coupling constant supporting a *β*-configuration. Additional signals in the δ_H_ 3.22–4.05 range were assignable to sugar protons.

**Figure 2:**
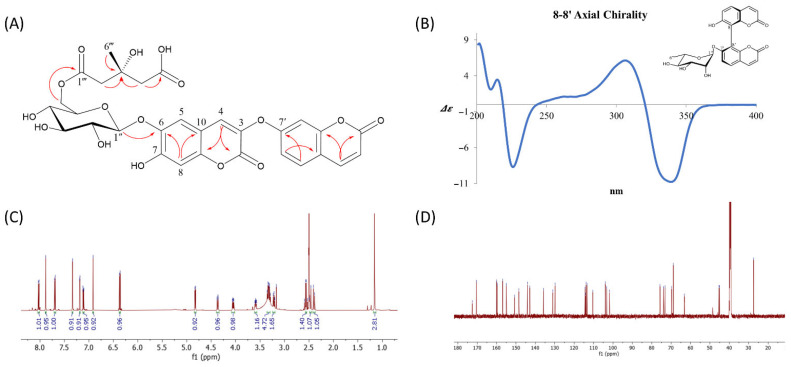
(**A**) Key HMBC correlations of the new compound **1**, highlighting the long-range proton–carbon couplings that were critical for establishing the connectivity between the coumarin skeleton, the glucose moiety, and the HMG side chain; (**B**) ECD spectrum of compound **4**, displaying characteristic cotton effects that facilitated the determination of its axial chirality and absolute configuration. (**C**) ^1^H NMR and (**D**) ^13^C NMR (**D**) spectra of new compound **1**.

The position of the glucose unit was suggested by an HMBC correlation from the anomeric proton (δ_H_ 4.83) to C-6 of the coumarin scaffold (δ_H_ 142.9). The downfield shift of the glucose methylene protons at δ_H_ 4.37 and 4.05 (H-6″) and their HMBC correlation with a carbonyl carbon at δ_C_ 170.4 (C-1‴) indicated the attachment of a 3-hydroxy-3-methylglutaric acid (HMG) moiety. The HMG group displayed characteristic NMR features, including large geminal couplings for two methylene groups [δ_H_ 2.40 (1H, d, *J* = 15.0 Hz, 2‴a)/2.47 (1H, d, *J* = 15.0 Hz, 2‴b), δ_H_ 2.54 (1H, d, *J* = 14.2 Hz, 4‴a)/2.58 (1H, d, *J* = 14.2 Hz, 4‴b)], one quaternary carbon (δ_C_ 68.7, C-3’’’), and two carbonyl carbons (δ_C_ 170.4, C-1’’’; 172.5, C-5‴). HMBC correlations of both methylene groups (H-2‴ and H-4‴) with the quaternary carbon (C-3‴) and their respective adjacent carbonyl carbons (H-2‴/C-1‴, H-4‴/C-5‴) further confirmed the structure of the HMG moiety. These data established compound **1** as a linearly connected dimeric coumarin bearing a glucose unit, with the HMG moiety attached to the methylene group of the glucose. The sugar moiety was confirmed as D-(+)-glucose by comparing the retention time of its thiocarbamoyl-thiazolidine derivative with that of an authentic D-(+)-glucose standard via reverse-phase HPLC, following the method described by Tanaka et al. [10]. Although the stereochemistry at C-3 of the HMG group was not directly determined, it is known that naturally occurring 3-hydroxy-3-methylglutarate is biosynthesized via the acylation of HMG-CoA during terpenoid formation [20]. Based on this biosynthetic origin, the C-3 position is presumed to possess an *S*-configuration. Based on comprehensive spectroscopic analysis, the structure of compound **1** was identified as 2H-1-Benzopyran-2-one, 6-[[6-O-(4-carboxy-3-hydroxy-3-methyl-1-oxobutyl)-β-d-glucopyranosyl]oxy]-7-hydroxy-3-[(2-oxo-2H-1-benzopyran-7-yl)oxy], which is reported here for the first time as a naturally occurring compound. To establish the absolute configuration of six axially chiral compounds (**4**–**9**), ECD calculations were conducted. The ECD spectra of compound **4** (Figure 2B) showed a negative cotton effect at 330 nm, indicative of exiton coupling. Comparison with published data [15] led to the assignment of the P-configuration for the 8–8′ axial chirality. The absolute configurations of the remaining compounds (**5**–**9**) were similarly determined based on their ECD profiles.

#### Spectroscopic and Physical Characteristics of New Compound **1**

2H-1-Benzopyran-2-one,6-[[6-O-(4-carboxy-3-hydroxy-3-methyl-1-oxobutyl)-β-d-glucopyranosyl]oxy]-7-hydroxy-3-[(2-oxo-2H-1-benzopyran-7-yl)oxy] (**1**): Colorless amorphous powder; ${\left[\alpha \right]}_{D}^{\mathrm{25}}$ + 32.5 (c 0.2, MeOH); UV (MeOH) *λ*_max_ (log ε) 263 (3.17), 338 (3.57) nm; IR (KBr) *ν*_max_ 3340, 1730, 1620, 1510, 1270 cm^−1^; ECD (MeOH) λmax (Δ*ε*) 216 (−1.32), 243 (−0.47), 284 (−0.10), 315 (−0.51), 341 (0.24), 359 (−0.40) nm. ^1^H and ^13^C NMR data (Figure 2C,D, Table 1); HRESIMS *m*/*z* 643.1289 [M − H]^−^ (calcd for C_30_H_27_O_16_, 643.1299).

### 3.2. Metabolomics and MS/MS-Based Chemical Profiling of Three Different Parts of *Edgeworthia chrysantha*

**Figure 3:**
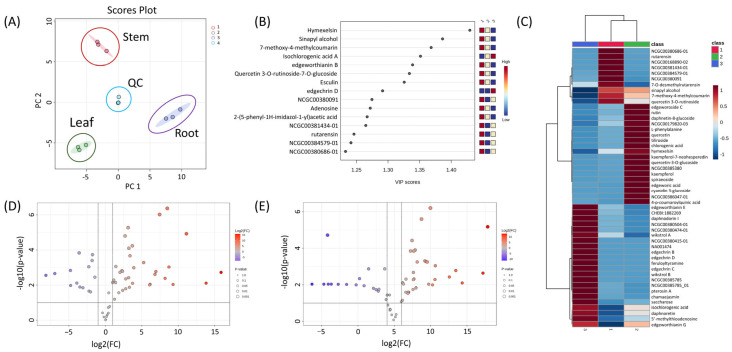
(**A**) PCA loading plot illustrating the metabolic distribution across three distinct plant parts (leaf, stem, and root) of *Edgeworthia chrysantha*; (**B**) PLS-DA scatter plot showing clear group separation based on metabolic profiles; (**C**) Heatmap visualization of normalized and clustered metabolite intensities across leaf, stem, and root samples, providing an overview of the relative abundance and grouping of structurally related compounds; (**D**) Volcano plot representing differential metabolite expression between leaf and stem tissues; (**E**) Volcano plot displaying differential metabolites between root and stem samples.

Metabolomics analysis was performed based on chemical profiling to better understand the metabolic differences among the three parts (leaf, stem, and root) of *E. chrysantha*. While the stem bark of this plant has long been used for papermaking and as a traditional remedy, and its constituents have been previously investigated, the roots and leaves remain largely unexplored in terms of their chemical composition. Principal component analysis (PCA) clearly distinguished the three parts, indicating distinct metabolic characteristics (Figure 3A). The scree plot revealed that the first two principal components accounted for 88.1% of the total variance, reflecting strong clustering and data separation (Figure S20A). Partial least squares discriminant analysis (PLS-DA) was used to identify key compounds responsible for group differentiation, as visualized in the scatter plot (Figure 3B). Hymexelsin showed the highest variable importance in projection (VIP) score, followed by sinapyl alcohol, 7-methoxy-4-methylcoumarin, isochlorogenic acid A, edgeworthianin B, and quercetin 3-*O*-rutinoside-7-*O*-glucoside. Hymexelsin, a coumarin glycoside composed of glucose and apiofuranose units, was most abundant in the stem, with smaller amounts detected in the leaf and only trace levels in the root. While the roots were rich in coumarin derivatives, these compounds were primarily dimers or trimers, lacking sugar moieties or containing only a single sugar unit. In contrast, monomeric coumarin glycosides such as hymexelsin and esculin were more concentrated in the stem than in the root. Relative concentrations of each compound were visualized in a heatmap following normalization and cluster analysis (Figure 3C). Additionally, three comparison groups were established: ‘leaf’ vs. ‘stem’, ‘root’ vs. ‘stem’, and ‘root’ vs. ‘leaf’. Based on PLS-DA (FC > 2.0 or < 0.5), a total of 32, 42, and 41 differential metabolites were identified from the ‘root’ vs. ‘leaf’, ‘leaf’ vs. ‘stem’, and ‘stem’ vs. ‘root’ comparisons, respectively, as shown in the volcano plot (Figure 3D,E and Figure S20B). As noted above, the root exhibited the highest concentrations of coumarin dimers/trimers, flavonoids, and edgechrin derivatives. Daphnoretin, a dimeric coumarin, was most abundant in the root. Daphnodorin, a spiro-type dimeric flavonoid, was also detected at high levels in the root. Although daphnoretin and daphnodorin belong to different structural classes, their names are similar as they were both first isolated from species of the genus *Daphne*. *Daphne*, *Edgeworthia*, and *Wikstroemia*, all members of the Thymelaeaceae family, are known to produce daphnodorin-type dimeric flavonoids [21,22]. Notably, this study also detected wikstrol A and B, flavonoid dimers originally identified in *Wikstroemia*, in the genus *Edgeworthia*. Both were most abundant in the roots of *E. chrysantha*.

Several antifungal compounds, including dimeric coumarins, dimeric flavonoids, and lignans, were found in high concentrations in the root. Daphnoretin was particularly abundant and has previously been reported to exhibit antifungal activity. In white clover (*Trifolium repens*), infection by soil-borne pathogens induced an increase in daphnoretin and other phenolics as part of the plant’s defense response [23]. Coumarins are known to be secreted from roots, influencing the rhizosphere microbiome [24]. Procyanidin A2, a dimeric flavonoid enriched in the root, also demonstrates antifungal effects [25]. Likewise, matairesinol, a lignan with established antifungal activity, was also abundant in the root [26]. These findings are consistent with notion that roots, being in contrast with soil-borne pathogens, produce and accumulate antifungal metabolites as a defense mechanism. In contrast, the stem contained higher levels of monomeric coumarin glycosides and monomeric flavonoid glycosides. In addition to hymexelsin, another coumarin glycoside, esculin, was also most concentrated in the stem. A distinguishing feature of the stem was its abundance of edgeworthianin derivatives. Edgeworthianins belong to the macrocyclic daphnane orthoesters (MDOs) family and feature a unique 1-alkyl side chain derived from a C14 aliphatic backbone, which is an uncommon structural motif in nature [9]. Furthermore, sinapyl alcohol, the second-highest VIP-scored compound in PLS-DA, was most abundant in the stem. Sinapyl alcohol is a monolignol that polymerizes to form S-rich lignin. It enhances hydraulic safety and forms a physical barrier to pathogen ingress [27]. The leaf showed higher concentrations of both aglycone and glycosylated flavonoids compared to the stem and root. Flavonoids are known to accumulate in the epidermal layers of leaves, where they absorb UV-B and UV-A radiation, thereby protecting chloroplasts and other photosynthetic components from photooxidative damage [28]. High levels of quercetin, quercetin-3-*O*-glycoside, kaempferol, and kaempferol-7-*O*-neohesperidin in the leaves of *E. chrysantha* may contribute not only to UV protection but also to reactive oxygen species (ROS) scavenging. By scavenging H_2_O_2_ and modulating NADPH oxidase (RBOH) activity, these flavonols regulate the magnitude and duration of ROS signaling, which in turn modulates guard cell signaling and stomatal aperture, ultimately influencing transpiration and drought responses [29]. Some coumarin glycosides were also found in the leaves. Interestingly, edgeworic acid, was most abundant in the leaves, suggesting that monomeric coumarins may combine with phenylpropanoid precursor specifically in leaf tissues. Coumarins were broadly distributed throughout *E. chrysantha*. Iron deficiency, common in alkaline, compacted, or phosphorus-rich soils, can upregulate genes like F6′H1, S8H, and CYP82C4 leading to enhanced coumarin biosynthesis in roots [30]. Transporter genes such as PDR9/ABCG37 are subsequently induced to secrete aglycone coumarins into the rhizosphere for iron mobilization [31]. During this process, many of the root-synthesized coumarins are converted into glycosylated forms and transported to the aerial parts via the xylem. Glycosylation enhances solubility and reduces toxicity, allowing for effective long-distance transport.

**Figure 4:**
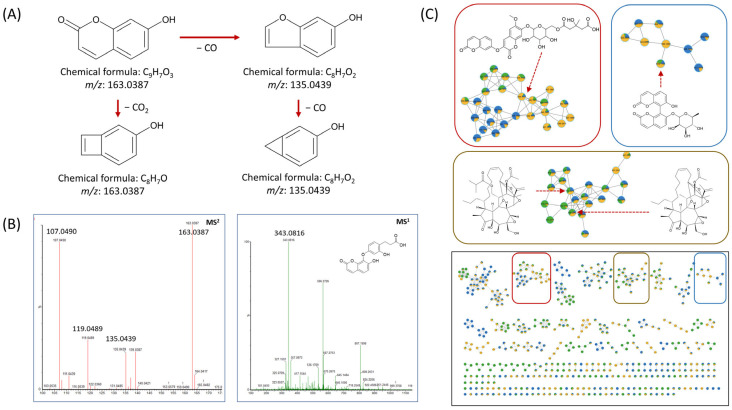
(**A**) Mass fragmentation patterns and their corresponding chemical structures; (**B**) MS1 and MS2 spectra of edgeworic acid, illustrating the precursor ion and key product ions resulting from characteristic bond cleavages.; (**C**) Cluster visualization from feature-based molecular networking (FBMN) analysis. Each differently colored box represents a distinct class of derivatives. Each node represents a detected ion, with edges indicating spectral similarity based on MS/MS fragmentation patterns. Structurally related metabolites, such as dimeric coumarins and flavonoid glycosides, formed distinct clusters, facilitating compound annotation and classification.

Fragmentation patterns provide deeper insights into compound structures. For example, edgeworic acid exhibited a protonated precursor ion at *m*/*z* 343.0816 (C_18_H_15_O_7_). Loss of ferulic acid moiety yielded a fragment ion at *m*/*z* 163.0387 (C_9_H_7_O_3_), followed by ketone loss to generate *m*/*z* 135.0439 (C_8_H_7_O_2_), and subsequent CO loss produced *m*/*z* 107.0496 (C_7_H_6_O) (Figure 4A,B). In negative ion mode, the deprotonated precursor was observed at *m*/*z* 341.0665 (C_18_H_13_O_7_), confirming the exact mass of 342.0739 (C_18_H_14_O_7_). A total of 64 compounds were annotated through UPLC-qTOF MS/MS-based chemical profiling. Among them, nine compounds (**1**–**9**) were subsequently isolated and identified to validate the MS/MS-based annotations. This combined approach strengthened the confidence level of the compound identification in the annotation of the results. Table 2 summarizes the retention time, *m*/*z* values, mass errors, and fragmentation patterns of each compound. Due to the length and complexity of some chemical names, simplified molecular input line entry system (SMILES) codes are used in Table 2, with the corresponding full chemical names provided in Supplementary Table S1. Structurally related compounds exhibiting similar fragmentation patterns were grouped and visualized in a molecular network (Figure 4C).

### 3.3. Bioassay and Molecular Docking Analysis

**Figure 5:**
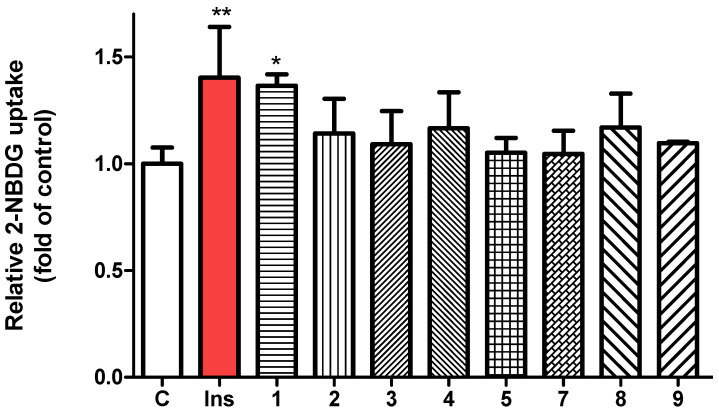
Evaluation of glucose uptake in 3T3-L1 adipocytes using the fluorescent glucose analog 2-NBDG, with insulin (100 nM) as positive control and isolated compounds **1**–**9** (each 20 µM) as test samples. Fluorescence intensity was measured at excitation/emission wavelengths of 450/535 nm. Data are presented as mean ± SD. * *p* < 0.05, ** *p* < 0.01 versus control.

To evaluate the glucose uptake activity of compounds **1**–**9**, a 2-NBDG uptake assay was performed using insulin as the positive control. Following treatment with each compound at a concentration of 20 µM, fluorescence intensity was measured using a microplate reader. Among the tested compounds, compound **1** exhibited the most pronounced enhancement of 2-NBDG uptake in 3T3-L1 adipocytes (Figure 5). Cell viability was assessed using the MTT colorimetric assay, which confirmed that all compounds (**1**–**9**) maintained cell viability without significant cytotoxicity at 20 µM (Figure S21). Glucose uptake is tightly regulated by a complex network of intracellular signaling pathways, with AMP-activated protein kinase (AMPK) serving as a key regulator. AMPK acts as a cellular energy sensor, typically activated by increased AMP/ATP ratios, but it can also be stimulated through allosteric mechanisms [32]. One such mechanism involves activation at the allosteric drug and metabolite (ADaM) site, located at the interface of the α-subunit kinase domain and the β-subunit carbohydrate-binding module (CBM). This activation enhances glucose transporter translocation to the plasma membrane, thereby promoting glucose uptake independently of insulin [33].

**Figure 6:**
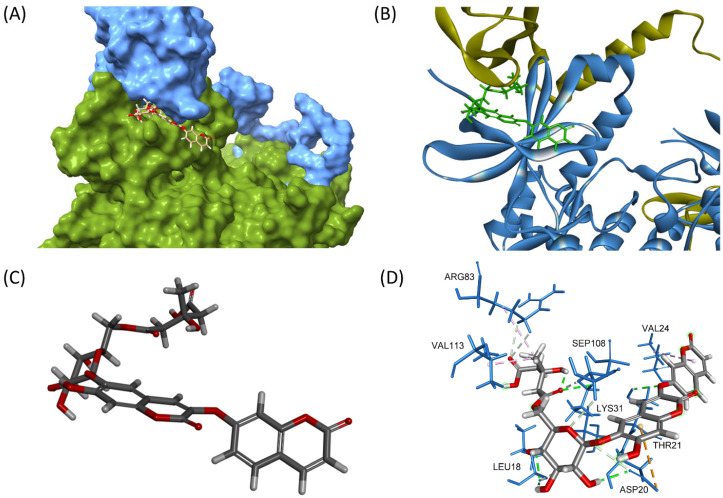
(**A**) Three-dimensional visualization of compound **1** bound to the allosteric site of AMPK, located at the interface between the α-subunit kinase domain (green) and the β-subunit CBM (blue). (**B**) Overlay of AMPK structure with compound **1** (green), highlighting its orientation in the allosteric pocket. (**C**) Molecular conformation of compound **1**, showing its coumarin dimer core, β-D-glucose, and HMG group positioned for optimal interactions. (**D**) Three-dimensional interaction diagram depicting key hydrogen bonds and hydrophobic contacts between compound **1** and surrounding AMPK residues.

To explore whether compound **1** activates AMPK via this allosteric mechanism, molecular docking studies were conducted. The crystal structure 5ISO was selected for molecular docking, as it contains a co-crystallized ligand (compound 991) at the ADaM site and has been validated by previous studies [12]. In addition to compound 991, another known allosteric activator, PF-06409577, was included for comparison. Compound **1** docked into the AMPK allosteric site with a CDocker energy of −26.17 kcal/mol and an interaction energy of −69.35 kcal/mol (Figure 6). Key interactions included conventional hydrogen bonds between Lys31 and the carbonyl group adjacent to the glycosidic linkage (2.63 Å), Thr21 and the ether bridge linking the two coumarin units (2.38 Å), and Leu18 and the glucose moiety (2.05 and 2.10 Å). A π-alkyl interaction was observed between Val24 and the coumarin ring (4.87 Å), while alkyl interactions involved Val113, Arg83, and the methyl group of the HMG moiety. Furthermore, Asp20 formed both a π-anion interaction with the coumarin ring (4.60 Å) and a conventional hydrogen bond with the glucose unit (2.22 Å). When compared with reference compounds, compound **1** showed favorable binding characteristics. Compound 991 yielded a CDocker energy of −36.07 kcal/mol and an interaction energy of −63.56 kcal/mol, while PF-06409577 showed a CDocker energy of −22.93 kcal/mol and an interaction energy of −47.76 kcal/mol. The bioactivity-related structural feature of compound **1** is its linear linkage containing both a carbonyl group and a phenyl ring, which is a characteristic similar to that of compound 991. These docking results correlate well with the observed glucose uptake activity, supporting the potential of compound **1** as an allosteric AMPK activator.

## 4. Conclusions

Distinct chemical profiles were observed among the leaf, stem, and root of *Edgeworthia chrysantha*, with the root particularly enriched in dimeric coumarins and flavonoids. Furthermore, the occurrence of wikstrols and daphnodorin-type dimers in *Edgeworthia* reinforces chemotaxonomic connections within Thymelaeaceae. Guided by metabolomic and bioactivity analyses, nine compounds (**1**–**9**) were isolated from the root, including one newly identified coumarin glycoside. Among these, compound **1** exhibited the strongest glucose uptake-stimulating activity without inducing cytotoxicity. Molecular docking analysis revealed that compound **1** binds favorably to the allosteric ADaM site of AMPK, suggesting a mechanism that may promote glucose uptake independently of insulin. While this study provides a comprehensive chemical and metabolomic profile of *E. chrysantha* and identifies compounds with glucose uptake-promoting activity, further studies, including in vivo experiments, are necessary to validate its anti-diabetic effects and explore additional bioactivities. Nonetheless, our findings support the anti-diabetic potential of *E. chrysantha*, particularly its root-derived constituents. Overall, this study highlights the therapeutic relevance of *E. chrysantha* as a promising source of novel AMPK-activating agents.

## Figures and Tables

**Table 1:** ^1^H- and ^13^C-NMR spectroscopic data of compound **1** in DMSO-*d*_6_^a^.

Position	1 ^b^	
	*δ*_H_ (*J* in Hz)	*δ*_C_
2	-	156.9
3	-	135.8
4	7.88 (1H, s)	131.0
5	7.34 (1H, s)	114.2
6	-	142.9
7	-	148.5
8	6.91 (1H, s)	103.2
9	-	150.7
10	-	110.4
2′	-	160.0
3′	6.37 (1H, d, 9.4)	113.8
4′	8.03 (1H, d, 9.6)	144.0
5′	7.70 (1H, d, 8.7)	129.9
6′	7.11 (1H, dd, 8.7, 2.4)	113.3
7′	-	159.7
8′	7.19 (1H, d, 2.5)	103.9
9′	-	155.0
10′	-	114.4
Glc		
1″	4.83 (1H, d, 7.4)	101.8
2″	3.36–3.28 (1H, m)	75.7
3″	3.36–3.28 (1H, m)	73.1
4″	3.22 (1H, m)	69.7
5″	3.59 (1H, dd, 9.1, 6.3)	74.0
6″	4.37 (1H, dd, 11.9, 2.0)	63.1
	4.05 (1H, dd, 11.9, 6.3)	
HMG		
1‴	-	170.4
2‴	2.58 (1H, d, 14.2)	45.1
	2.54 (1H, d, 14.2)	
3‴	-	68.7
4‴	2.47 (1H, d, 15.0) 2.40 (1H, d, 15.0)	45.3
5‴	-	172.5
6‴	1.16 (3H, s)	27.5

^a^ Assignments were based on COSY, HSQC, and HMBC experiments. ^b 1^H and ^13^C NMR spectra were acquired at 600 and 150 MHz, respectively.

**Table 2:** UPLC-qTOF MS/MS, combined with GNPS-based molecular networking, enabled the annotation of 64 metabolites from *E. chrysantha*.

no	rt	*m*/*z*	Theoretical Mass ^a^	Chemical Formula	Error [ppm]	Name ^b^	Fragment	Detected Part ^c^	Mode
1	1.14	365.1054	365.1059	C_12_H_22_O_11_	−1.36	Saccharose	204.0543, 203.0522, 185.0420	L, S, R	[M + Na]^+^
2	1.16	268.1042	268.1045	C_10_H_13_N_5_O_4_	−0.89	Adenosine	137.0635, 136.0616, 120.0376, 119.0351	L, S, R	[M + H]^+^
3	1.22	203.0809	203.0820	C_11_H_10_N_2_O_2_	−5.41	2-(5-phenyl-1H-imidazol-yl)acetic acid	157.0755, 130.0650, 128.0495, 103.0542	L, S, R	[M + H]^+^
4	1.27	384.1152	384.1155	C_14_H_17_N_5_O_8_	−0.88	S-N6-succinyladenosine	192.0510, 188.0551, 162.0766, 136.0620	L, S, R	[M + H]^+^
5	1.45	166.0861	166.0868	C_9_H_11_NO_2_	−3.61	Phenylalanine	121.0837, 113.0245 103.0535	L, S, R	[M + H]^+^
6	1.75	355.1025	355.1029	C_16_H_19_O_9_	−1.12	Chlorogenic acid	163.0388, 145.0282, 135.0439, 117.0334	L, S, R	[M + H]^+^
7	1.78	298.0969	298.0973	C_11_H_15_N_5_O_3_S	−1.63	5′-Methylthioadenosine	137.0633, 136.0616, 120.0392, 119.0352	L, S, R	[M + H]^+^
8	1.98	437.1459	437.1447	C_21_H_24_O_10_	2.57	CHEBI:182269	149.0594, 145.0647, 139.0388, 107.0487	L, S, R	[M + H]^+^
9	2.01	453.1392	453.1396	C_21_H_24_O_11_	−0.88	NCGC00385785	163.0387, 147.0440, 139.0388, 123.0440	L, S, R	[M + H]^+^
10	2.03	341.0869	341.0872	C_15_H_16_O_9_	−1.05	Esculin	179.0336, 151.0381, 133.0281, 123.0438	L, S, R	[M + H]^+^
11	2.06	205.0968	205.0977	C_11_H_12_N_2_O_2_	−0.01	Tryptophan	143.0725, 118.0649, 117.0577, 115.0541	L, S, R	[M + H]^+^
12	2.15	341.0868	341.0872	C_15_H_16_O_9_	−0.17	Daphnetin-8-O-glucoside	179.0337, 133.0283, 123.0440, 105.0335	L, S, R	[M + H]^+^
13	2.17	453.1394	453.1396	C_21_H_24_O_11_	−0.63	NCGC00385785-01	163.0385, 147.0444, 139.0385, 123.0442	S, R	[M + H]^+^
14	2.19	457.1344	457.1346	C_20_H_24_O_12_	−0.43	NCGC00380504-01	164.0420, 163.0388, 119.0489, 107.0490	L, S, R	[M + H]^+^
15	2.26	325.0921	325.0923	C_15_H_16_O_8_	−0.61	NCGC00384579-01	107.0490, 119.0490, 163.0387, 164.0422	L, S, R	[M + H]^+^
16	2.41	773.2151	773.2140	C_33_H_40_O_21_	1.37	Quercetin-3-O-glc-1-3-rham-1-6-glucoside	303.0503, 157.0134, 121.0142	L, S, R	[M + H]^+^
17	2.45	471.1497	471.1502	C_21_H_26_O_12_	−1.17	NCGC00380091	163.0388, 113.0422	L, S, R	[M + Na]^+^
18	2.48	627.1534	627.1561	C_27_H_30_O_17_	−4.35	Luteolin-3-O-glc-(1-6)-glucopyranoside	287.0543, 221.0435, 213.0135	L, S	[M + H]^+^
19	2.83	177.0544	177.0551	C_10_H_10_O_4_	−4.34	trans-Ferulic acid	134.0357, 117.0332, 105.0332	L, S, R	[M + H]+
20	2.92	521.2022	521.2022	C_26_H_34_O_12_	−0.17	NCGC00381098	163.0751, 131.0490, 103.0541	L, S, R	[M + H]+
21	2.94	757.2204	757.2191	C_33_H_40_O_20_	1.68	Cyanidin 3-O-rutinoside	287.0553, 225.0584, 189.0601	L, S, R	[M + H]^+^
no	rt	*m*/*z*	Theoretical Mass ^a^	Chemical Formula	Error [ppm]	Name ^b^	Fragment	Detected Part ^c^	Mode
22	2.98	363.1805	363.1807	C_20_H_26_O_6_	−0.72	NCGC00380686-01	147.0439, 123.0440, 119.0491, 105.0698	L, S, R	[M + H]^+^
23	3.16	773.2145	773.2140	C_33_H_40_O_21_	0.59	Quercetin 3-O-rutinoside-7-O-glucoside	303.0503, 157.0354, 113.0684	L, S, R	[M + H]^+^
24	3.17	289.0715	289.0712	C_15_H_12_O_6_	0.98	Dihydrokaempferol	215.0704, 149.0230, 123.0451, 121.0293	L, S, R	[M + H]^+^
25	3.42	385.1133	385.1134	C_17_H_20_O_10_	−0.25	NCGC00168890-02	223.0598, 208.0362, 189.0257, 161.0309	L, S, R	[M + H]^+^
26	3.44	191.07	191.0708	C_11_H_10_O_3_	−4.18	7-methoxy-4-methylcoumarin	133.0281, 118.0413, 115.0541, 103.0539	L, S, R	[M + H]^+^
27	3.47	193.0855	193.0864	C_11_H_14_O_4_	−4.6	Sinapyl alcohol	118.0415, 115.0549, 105.0692, 103.0537	L, S, R	[M + Na]^+^
28	3.53	345.1696	345.1702	C_20_H_26_O_6_	−1.73	2,3-bis[(4-hydroxy-3-methoxyphenyl)methyl]butane 1,4-diol	137.0595, 131.0489, 122.0360, 103.0541	L, S, R	[M + Na]^+^
29	3.53	327.1593	327.1596	C_20_H_22_O_4_	−1.02	Isolicarin A	137.0595, 131.0489, 122.0361, 103.0541	L, S, R	[M + H]^+^
30	3.59	339.1090	339.1079	C_16_H_18_O_8_	3.24	Coumaroyl quinic acid	221.1541, 123.0541	L, S, R	[M + H]^+^
31	3.61	611.1616	611.1612	C_27_H_30_O_16_	0.65	Rutin	303.0413, 151.1542, 111.0265	L, S, R	[M + H]^+^
32	3.70	303.0504	303.0504	C_15_H_10_O_7_	−0.26	Quercetin	229.0494, 201.0543, 137.0232	L, S, R	[M + H]^+^
33	3.73	465.1031	465.1033	C_21_H_20_O_12_	−0.44	Spiraeoside	303.0503, 229.0495, 153.0181	L, S, R	[M + H]^+^
34	3.79	577.1331	577.1346	C_30_H_24_O_12_	−2.60	Procyanidin A2	299.0526, 287.0556, 123.0436	L, S, R	[M + H]^+^
35	3.81	487.1445	487.1451	C_21_H_26_O_13_	−1.23	Hemexelsin	212.1354, 175.0424, 135.0347	L, S	[M + H]^+^
36	3.85	314.1392	314.1392	C_18_H_19_NO_4_	−0.10	Feruloyltyramine	145.0283, 121.0643, 117.0331, 103.0548	R	[M + H]^+^
37	3.95	595.1665	595.1663	C_27_H_30_O_15_	0.33	Kaempferol-7-neohesperidoside	287.0552, 213.0485, 189.0605	L, S, R	[M + H]^+^
38	3.95	287.0551	287.0555	C_15_H_10_O_6_	−1.61	Kaempferol	213.0540, 165.0173, 153.0178, 121.0278	L, S, R	[M + H]^+^
39	3.96	249.1483	249.1490	C_15_H_20_O_3_	−3.09	Pterosin A	165.0700, 141.0695, 115.0543	L, S, R	[M + H]^+^
40	3.97	469.1133	469.1134	C_24_H_20_O_10_	−0.21	Edgeworoside C	323.0554, 249.0547, 221.0593	L, S, R	[M + H]^+^
41	4.01	245.1167	245.1177	C_15_H_16_O_3_	−4.36	(1R)-3,8-Dimethyl-5,14-dioxatricyclo [10.2.1.0~2,6~]pentadeca-2(6),3,8,12(15)-tetraen-13-one	141.0701, 129.0689, 115.0539	L, S, R	[M + H]^+^
no	rt	*m*/*z*	Theoretical Mass ^a^	Chemical Formula	Error [ppm]	Name ^b^	Fragment	Detected Part ^c^	Mode
42	4.05	449.108	449.1083	C_21_H_20_O_11_	−0.86	NCGC00386047-01	287.0553, 165.0180, 153.0179	L, S, R	[M + Na]^+^
43	4.18	499.1235	499.1240	C_25_H_24_O_12_	−1.08	Isochlorogenic acid A	163.0387, 145.0283, 135.0437, 117.0331	L, S, R	[M + H]^+^
44	4.38	499.0882	499.0876	C_24_H_20_O_12_	1.20	Daphneretusin A	337.0352, 281.0456, 192.0060	L, S, R	[M + H]^+^
45	4.47	643.1289	643.1298	C_30_H_28_O_16_	−1.39	7-O-desmethylrutarensin	337.0349, 281.1045, 215.1426, 192.0057	L, S, R	[M − H]-
46	4.47	541.1135	541.1134	C_30_H_22_O_10_	0.18	Wikstrol A	431.1145, 289.1040, 295.0617, 151.0037	S, R	[M + H]^+^
47	4.47	541.1135	541.1134	C_30_H_22_O_10_	0.18	Wikstrol B	431.1143, 289.1045, 295.0622, 151.0042	S, R	[M + H]^+^
48	4.50	543.1290	543.1291	C_30_H_22_O_10_	−0.18	Daphnodorin I	281.0443, 225.0544, 153.0179	S, R	[M + H]^+^
49	4.55	545.1421	545.1447	C_30_H_24_O_10_	−4.7	NCGC00380415-01	137.0625, 136.0617, 119.0353	S, R	[M + H]^+^
50	4.57	543.1292	543.1291	C_30_H_22_O_10_	0.13	NCGC00380474-01	311.0555, 281.0448, 225.0546, 153.0182,	L, S, R	[M + H]^+^
51	4.60	1065.2229	1065.2242	C_60_H_42_O_19_	−1.22	Edgechrin B	431.1178, 261.1126, 217.1228	S, R	[M + H]+
52	4.65	343.0816	343.0817	C_18_H_14_O_7_	−0.29	Edgeworic acid	163.0387, 135.0439, 119.0489, 107.0470,	L, S, R	[M + H]+
53	4.74	643.1289	643.1299	C_30_H_28_O_16_	−1.55	Compound 1	603.2067, 543.1282, 455.1913	R,S	[M − H]-
54	4.98	659.1629	659.1612	C_31_H_30_O_16_	2.55	Rutarensin	353.0659, 338.0425, 179.0339, 127.0389	L, S, R	[M + H]+
55	5.00	595.1468	595.1451	C_30_H_26_O_13_	2.73	Tiliroside	119.0491, 147.0439, 287.0554	L, S, R	[M + H]+
56	5.16	613.0982	613.0980	C_32_H_22_O_13_	0.32	Edgeworoside B	437.0663, 393.0763, 161.0239	L, S, R	[M − H]-
57	5.27	643.1081	643.1085	C_33_H_24_O_14_	−0.62	7″-O-glc-triumbelletin	421.0597, 353.0153, 277.9570	L, S, R	[M − H]-
58	5.43	543.1281	543.1291	C_30_H_22_O_10_	−1.88	Chamaejasmin	231.0687, 153.0178, 121.0278, 107.0491	S, R	[M + H]+
59	5.60	1051.2457	1051.2461	C_60_H_42_O_18_	−0.38	Edgechrin C	479.6830, 398.1020, 347.2273	S, R	[M + H]+
60	5.81	359.1487	359.1494	C_20_H_22_O_6_	−1.94	Matairesinol	137.0594, 131.0486, 103.0544	S, R	[M + H]+
61	5.91	351.0542	351.0529	C_19_H_12_O_7_	3.70	Daphnoretin	311.0477, 179.0339, 163.0035	L, S, R	[M − H]-
62	5.92	1051.2457	1051.2461	C_60_H_42_O_18_	−0.38	Edgechrin D	479.6830, 398.1020, 347.2273	S, R	[M + H]+
63	6.05	321.0406	321.0399	C_18_H_10_O_6_	2.13	Edgeworin	252.1228, 165.0034, 135.0085	L, S, R	[M − H]-
64	7.85	329.1387	329.1389	C_19_H_20_O_5_	−0.36	NCGC00179820-03	229.0858, 213.0546, 185.0595	L, S, R	[M + H]+

^a^ Theoretical masses were calculated based on the chemical formulas and ionization modes ^b^ Compounds with lengthy names were assigned SMILES codes; their full names are provided in the Supplementary Materials ^c^ L: leaf; S: stem; R: root.

## Data Availability

The data that support the findings of this study are available from the corresponding author upon reasonable request.

## Supplementary Materials

The following supporting information can be downloaded at: https://www.mdpi.com/article/10.3390/nu17162684/s1.

## Abbreviations

The following abbreviations are used in this manuscript:

AMPK	AMP-activated protein kinase
HMBC	Heteronuclear multi-bond correlation
HPLC	High performance liquid chromatography
HRESIMS	High-resolution electrospray ionization mass spectrometry
HSQC	Heteronuclear single quantum coherence
HMG	3-hydroxyl-e-methyl glutaroyl moiety
LC-MS	Liquid chromatography-mass spectrometry
NMR	Nuclear magnetic resonance
TLC	Thin layer chromatography
