# Supplementary material for: Chemical Profiling and UPLC-qTOF-MS/MS-Based Metabolomics of Three Different Parts of Edgeworthia chrysantha and Identification of Glucose Uptake-Enhancing Compounds
Source: Nutrients. 2025 Aug 19;17(16):2684. doi: 10.3390/nu17162684 (PMC12389323; doi:10.3390/nu17162684)
Supplement: Supplementary file 1 [file nutrients-17-02684-s001.zip › nutrients-3809399-supplementary.pdf]

Supplementary Material

Chemical Profiling and UPLC-qTOF-MS/MS-Based Metabolomics of Three Different Parts of *Edgeworthia chrysantha* and Identification of Glucose Uptake-Enhancing Compounds

Jin-Pyo An [1], Sohee Han [1], Van-Hieu Mai [1], Jorge-Eduardo Ponce-Zea [1], Gi Hyeon Seong [1], Thi-Kim-Quy Ha [1,2], Won Keun Oh [1,*]

[1] Research Institute of Pharmaceutical Sciences, College of Pharmacy, Seoul National University, Seoul 151-742, Republic of Korea; ntopjp77@snu.ac.kr (J.P.A); sohee.hn@snu.ac.kr (S.H); maihieu@snu.ac.kr (V.H.M); jepz210689@snu.ac.kr (J.E.P); ghseong00@snu.ac.kr (G.H.S); wkoh1@snu.ac.kr (W.K.O)

[2] College of Natural Sciences, Cantho University, Campus II, Can Tho 90000, Vietnam; htkquy@ctu.edu.vn (T.K.Q.H)

[*] To whom correspondence should be addressed. Tel & Fax: +82-02-880-7872. E-mail: wkoh1@snu.ac.kr

Table of Contents

Figure S1. Fractionation scheme of *Edgeworthia chryantha* roots…..…………………..……3
Figure S2. ^{1}H NMR spectrum (DMSO-d_6, 600 MHz) of compound **1**……………………..……4
Figure S3. ^{1}C NMR spectrum (DMSO-d_6, 150 MHz) of compound **1**……………………..……4
Figure S4. HSQC spectrum (DMSO-d_6, 600 MHz) of compound **1**……………….……………5
Figure S5. HMBC spectrum (DMSO-d_6, 600 MHz) of compound **1**………...……………………5
Figure S6. ^{1}H and ^{13}C NMR spectrum (DMSO-d_6, 600/150 MHz) of compound **2**……………..6
Figure S7. ^{1}H and ^{13}C NMR spectrum (DMSO-d_6, 500/125 MHz) of compound **3**……………..7
Figure S8. ^{1}H and ^{13}C NMR spectrum (DMSO-d_6, 600/150 MHz) of compound **4**……………..8
Figure S9. ECD spectrum of compound **4**………………………………………………………..9
Figure S10. ^{1}H and ^{13}C NMR spectrum (DMSO-d_6, 600/150 MHz) of compound **5**……………10
Figure S11. ECD spectrum of compound **5**……………………………………………………11
Figure S12. ^{1}H and ^{13}C NMR spectrum (DMSO-d_6, 600/150 MHz) of compound **6**……………12
Figure S13. ECD spectrum of compound **6**……………………………………………………..13
Figure S14. ^{1}H and ^{13}C NMR spectrum (MeOD-d_4, 600/150 MHz) of compound **7**……………14
Figure S15. ECD spectrum of compound **7**……………………………………………………15
Figure S16. ^{1}H and ^{13}C NMR spectrum (MeOD-d_4, 400/100 MHz) of compound **8**……………16
Figure S17. ECD spectrum of compound **8**……………………………………………………17
Figure S18. ^{1}H and ^{13}C NMR spectrum (DMSO-d_6, 600/150 MHz) of compound **9**……………18
Figure S19. ECD spectrum of compound **9**……………………………………………………19
Figure S20. Scree plot of PCA (A). Volcano plot of root vs leaf (B)..………………………20
Figure S21. Effects of isolated compounds on cytotoxicity in 3T3-L1 adipocytes..…………21
Table S1. Structure and SMILES code of labeled compounds…..………………………22

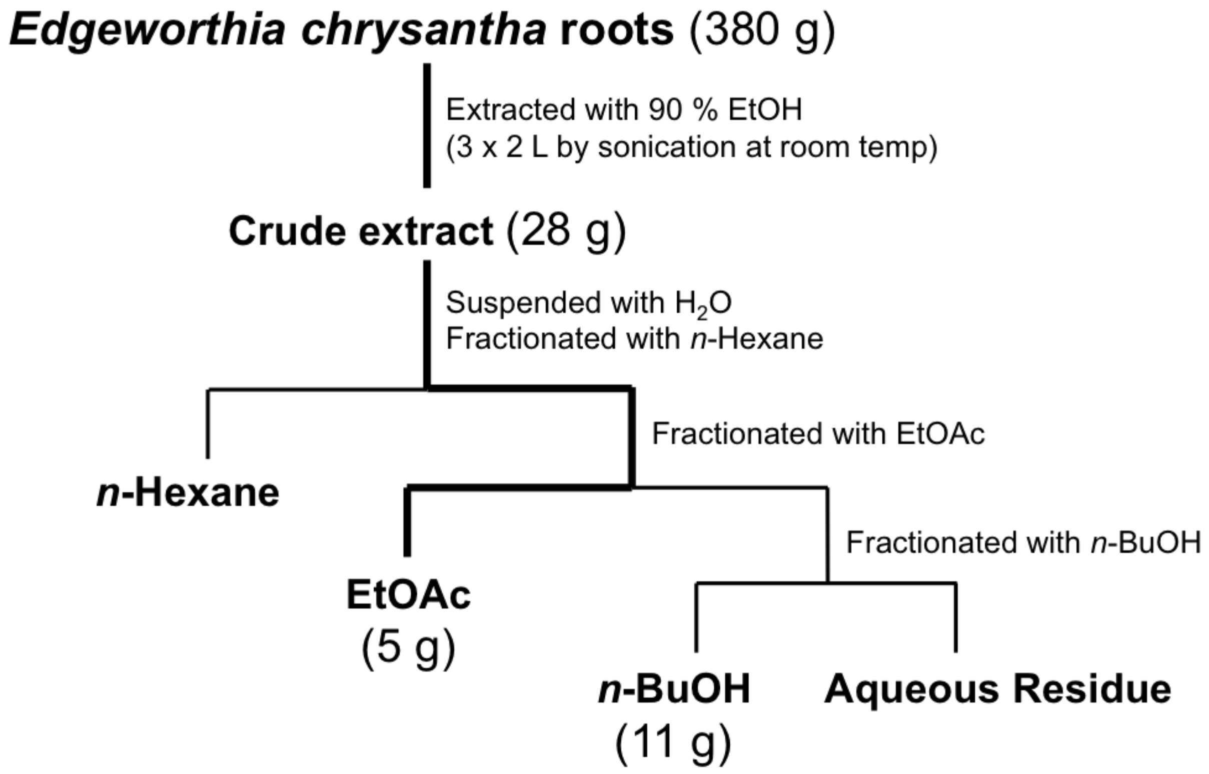

Figure S1. Fractionation scheme of *Edgeworthia chryantha* roots using liquid-liquid extraction method.

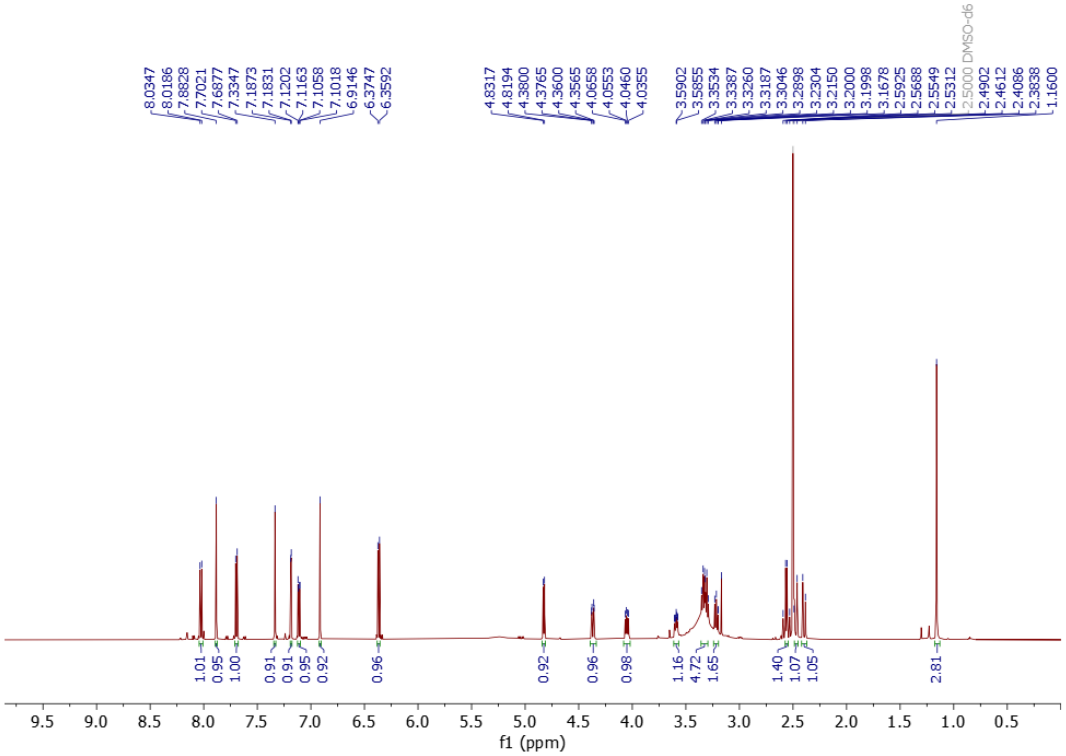

Figure S2. ^{1}H NMR spectrum (DMSO-d_6, 600 MHz) of compound **1.**

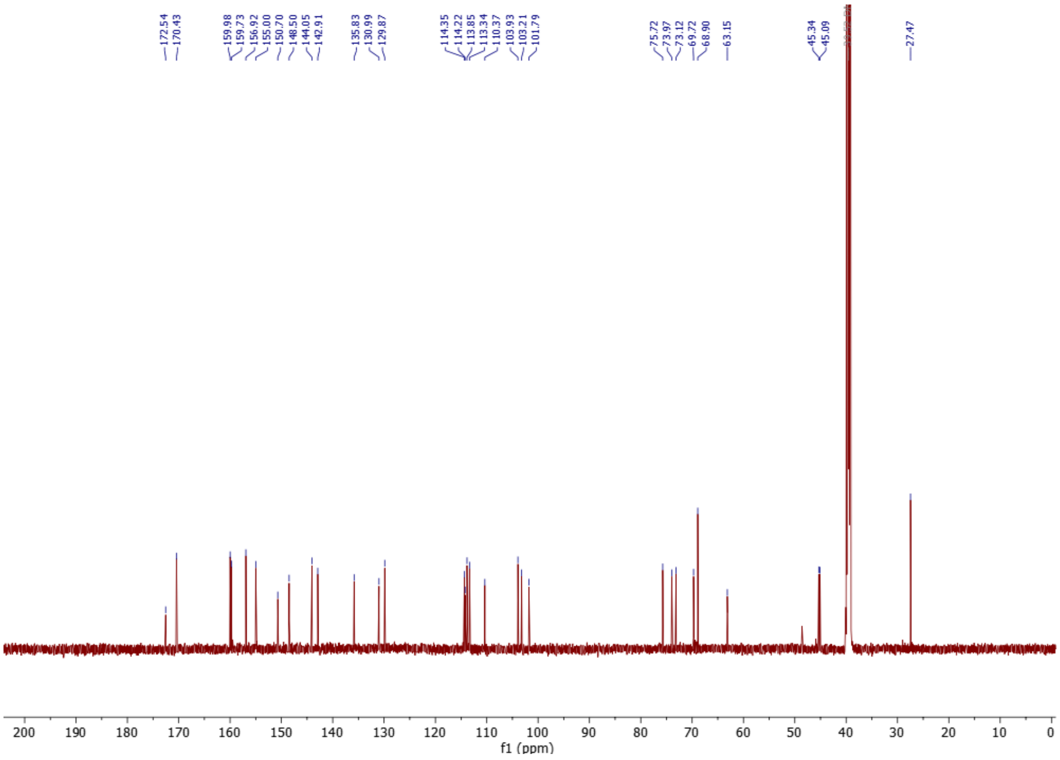

Figure S3. ^{13}C NMR spectrum (DMSO-d_6, 150 MHz) of compound **1.**

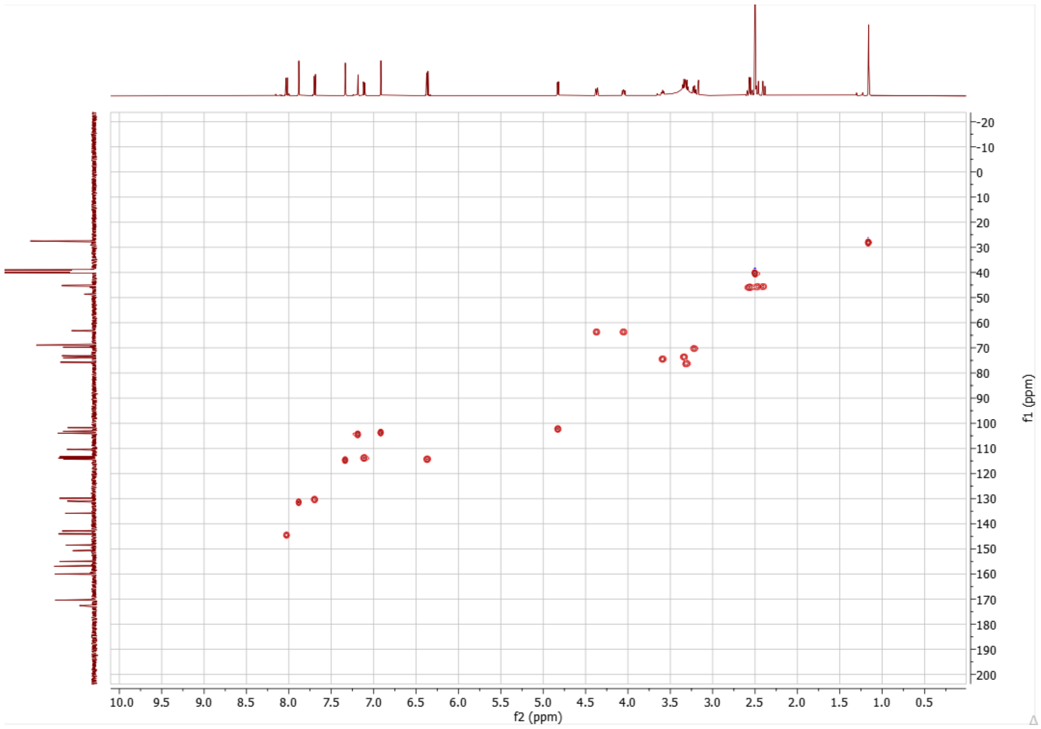

Figure S4. HSQC spectrum (DMSO-d_6, 600 MHz) of compound **1.**

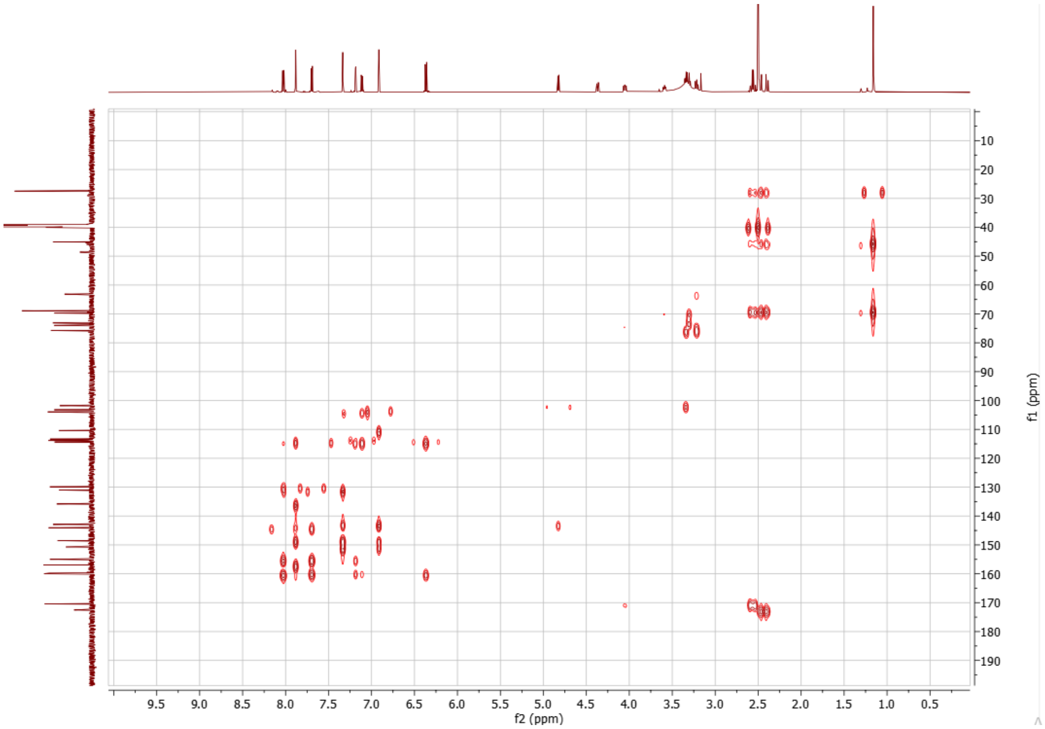

Figure S5. HMBC spectrum (DMSO-d_6, 600 MHz) of compound **1.**

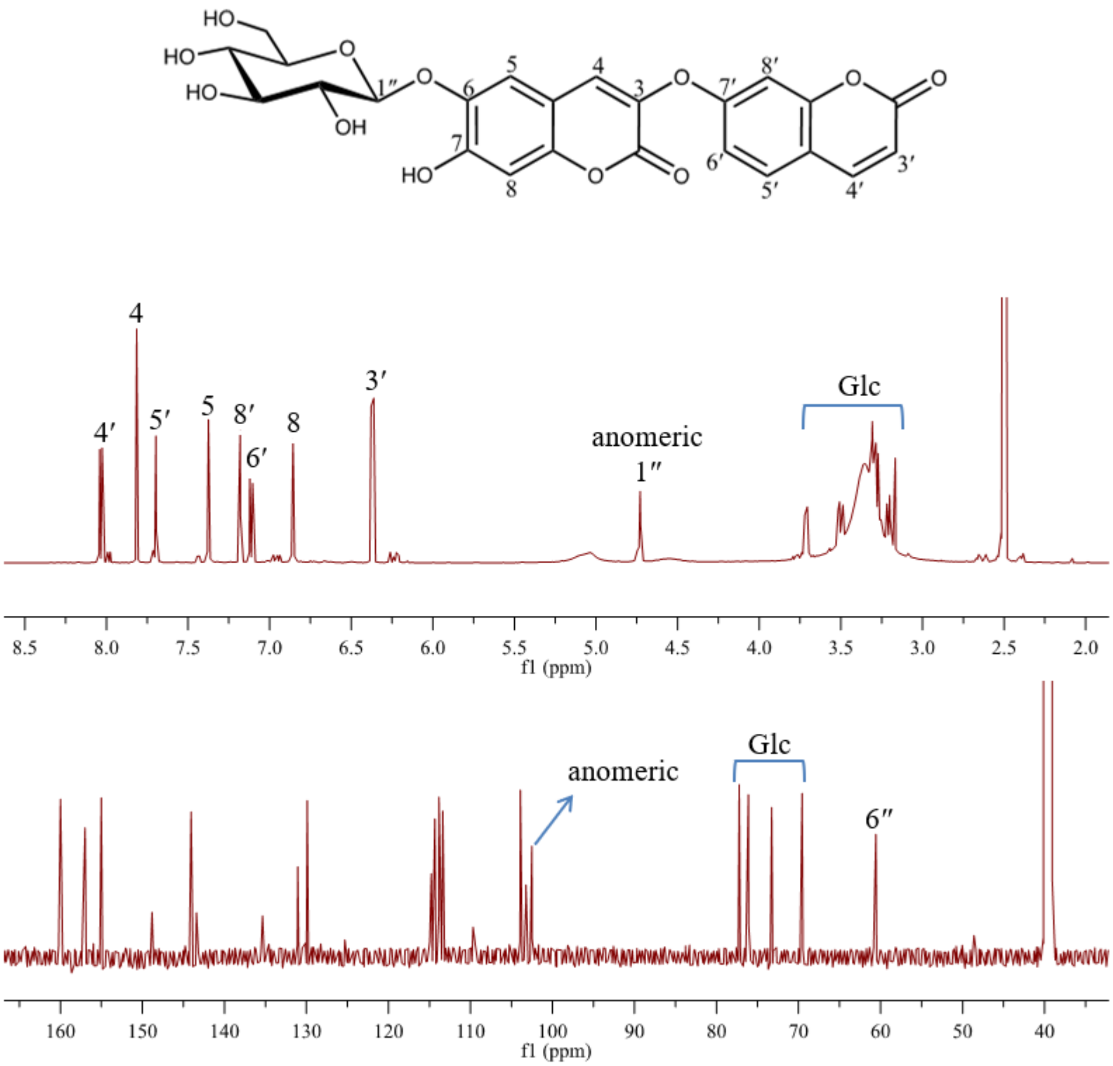

Figure S6. ^{1}H and ^{13}C NMR spectrum (DMSO-d_6, 600/150 MHz) of compound **2.**

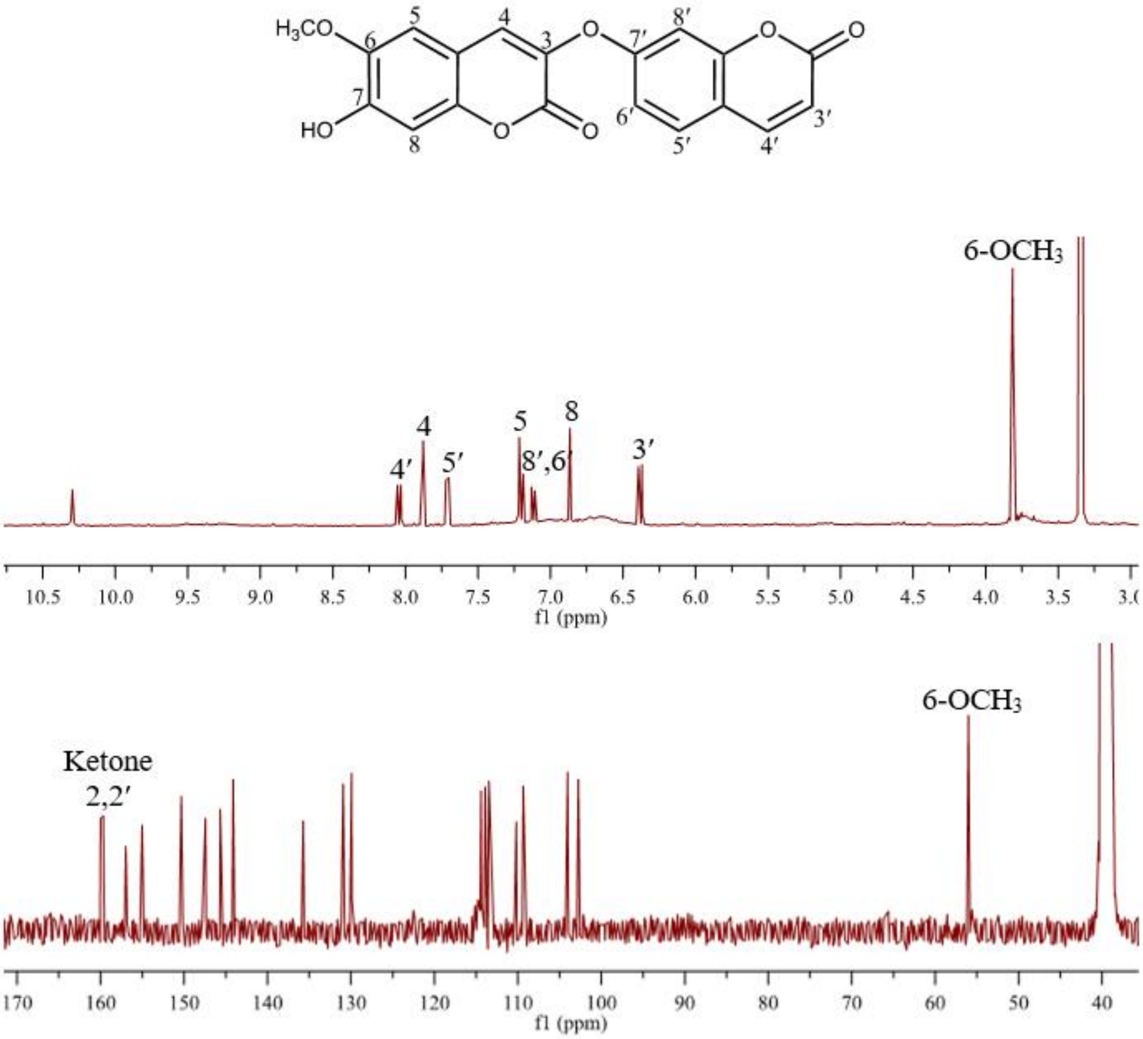

Figure S7. ^{1}H and ^{13}C NMR spectrum (DMSO-d_6, 600/150 MHz) of compound **3.**

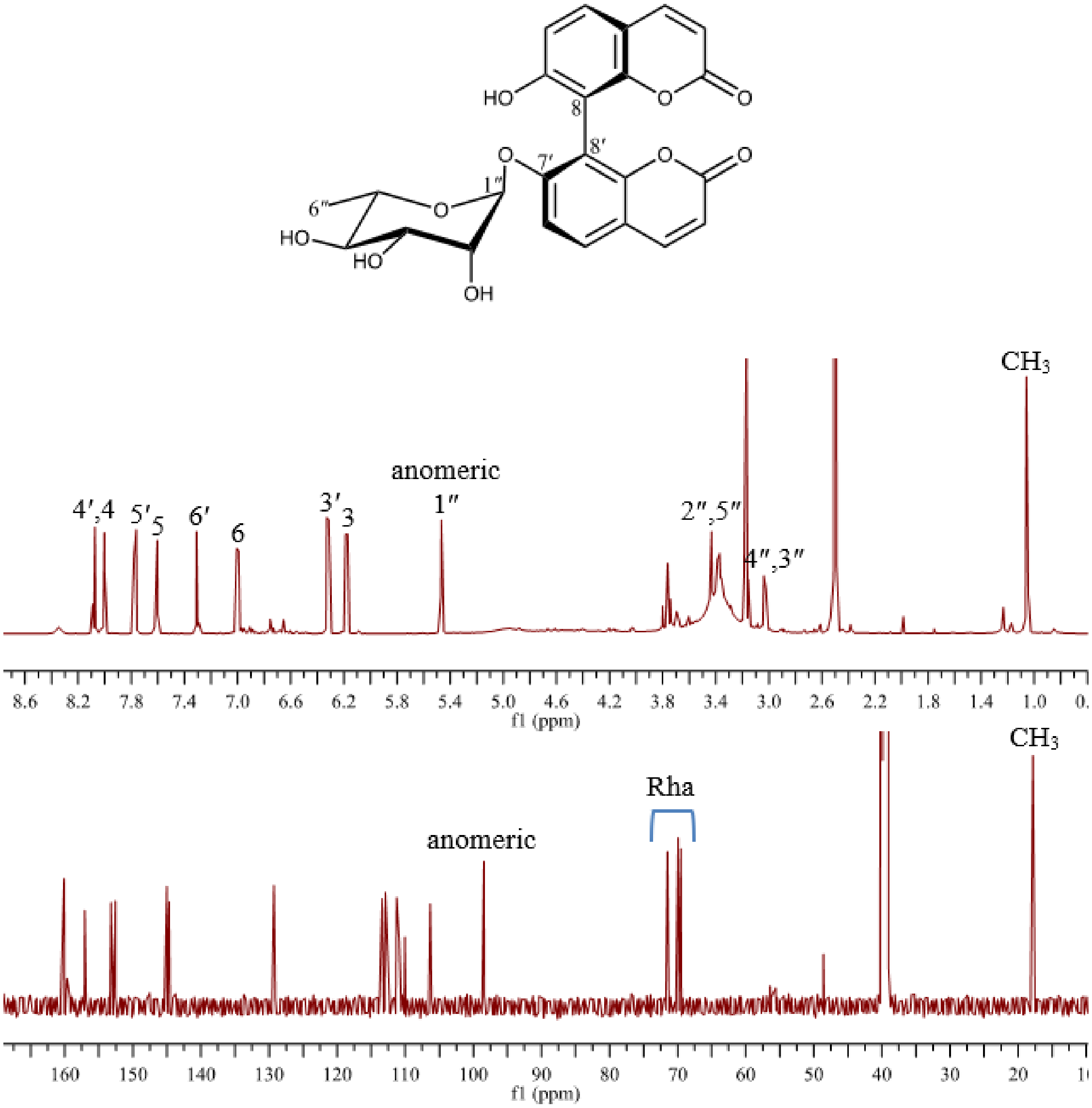

Figure S8. ^{1}H and ^{13}C NMR spectrum (DMSO-d_6, 600/150 MHz) of compound **4.**

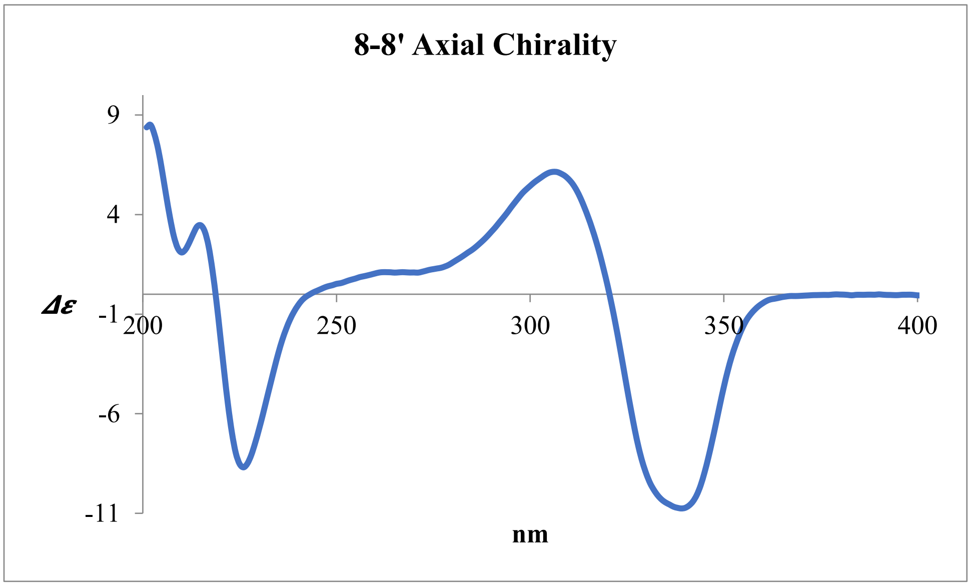

Figure S9. ECD spectrum of compound **4.**

$[\alpha]_D^{20}$= -59.0° (c 0.08, MeOH)

UV (MeOH) λmax (log ε) 286 (2.89), 324 (2.83), 337 (2.82) nm

ECD (MeOH) λmax (*Δε*) 227 (-0.16), 279 (-0.11), 298 (-0.02), 333 (-0.06) nm

HRESIMS m/z 467.0972 $[M - H]^-$ (calcd for $C_{24}H_{19}O_{10}$, 467.0978) .

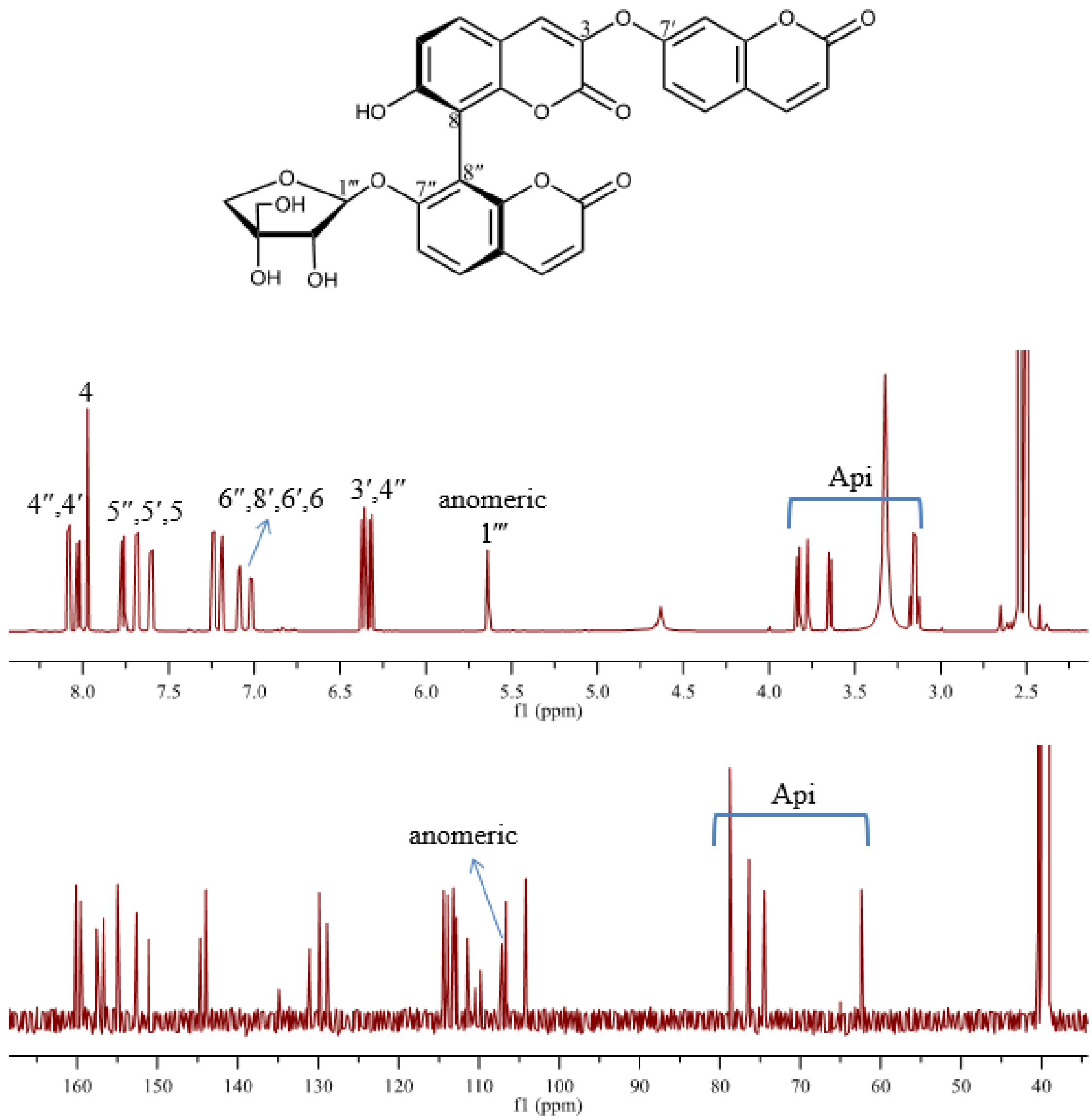

Figure S10. ^{1}H and ^{13}C NMR spectrum (DMSO-d_6, 600/150 MHz) of compound **5.**

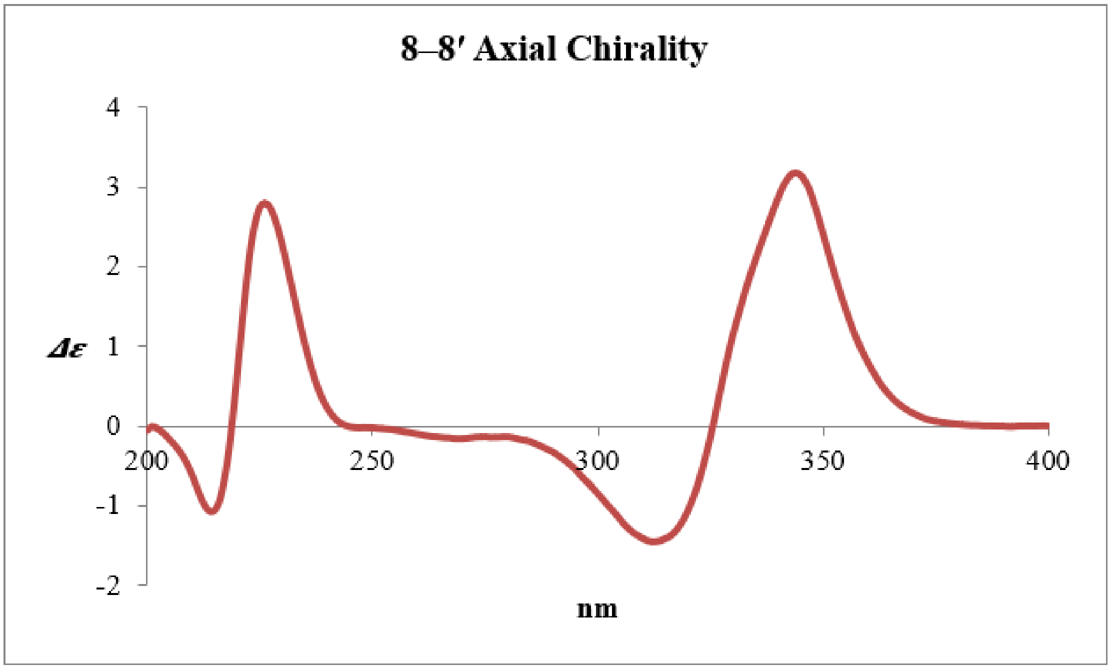

Figure S11. ECD spectrum of compound **5.**

$[\alpha]_D^{20}$= -96.2° (c 0.1, MeOH)

UV (MeOH) λmax (log ε) 207 (3.69), 325 (3.49) nm

ECD (MeOH) λmax (*Δε*) 214 (-1.08), 226 (2.79), 312 (-1.46), 344 (3.17) nm

HRESIMS m/z 613.0980 $[M - H]^-$ (calc. for $C_{32}H_{21}O_{13}$, 613.0982).

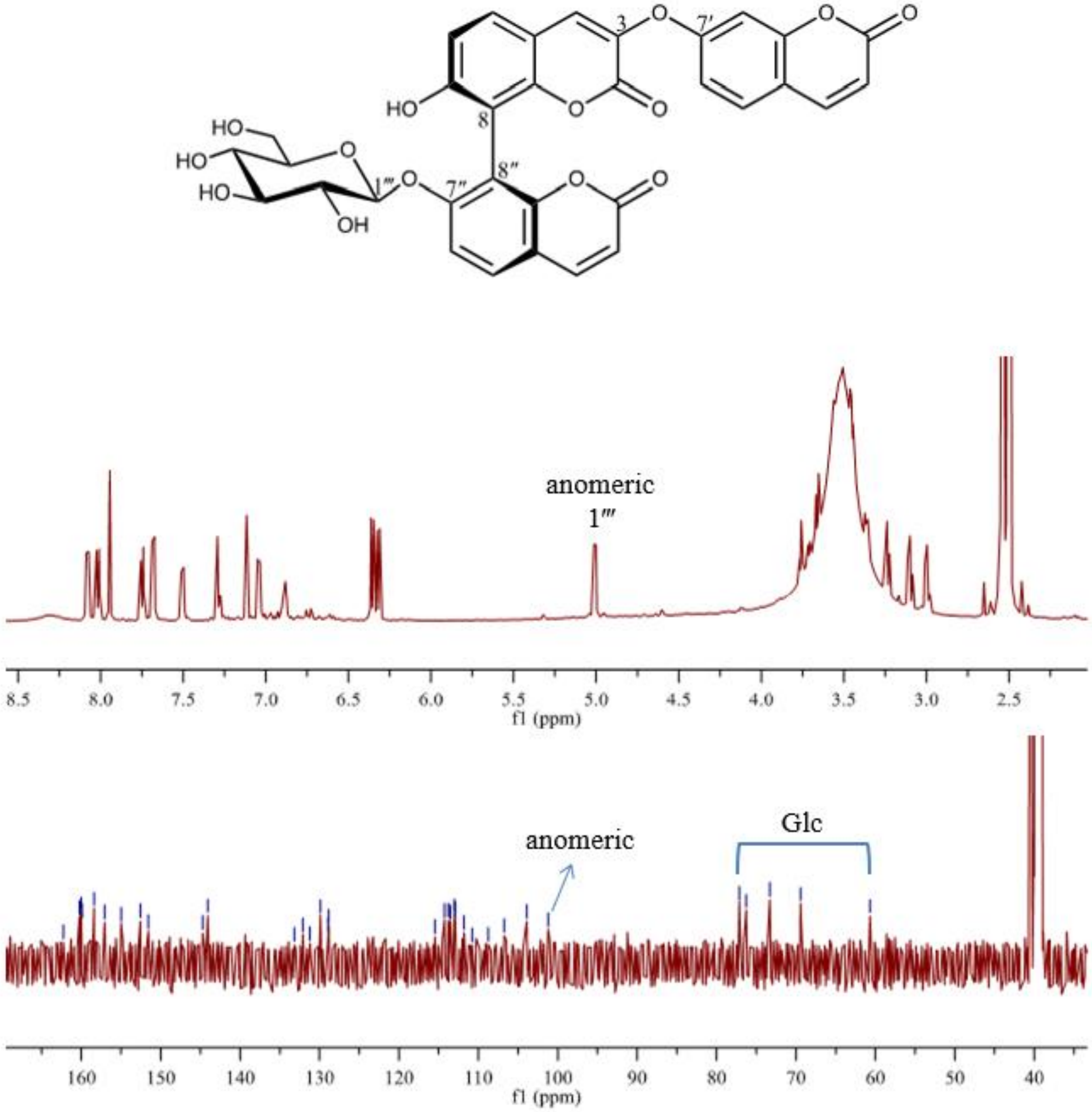

Figure S12. ^{1}H and ^{13}C NMR spectrum (DMSO-d_6, 600/150 MHz) of compound **6.**

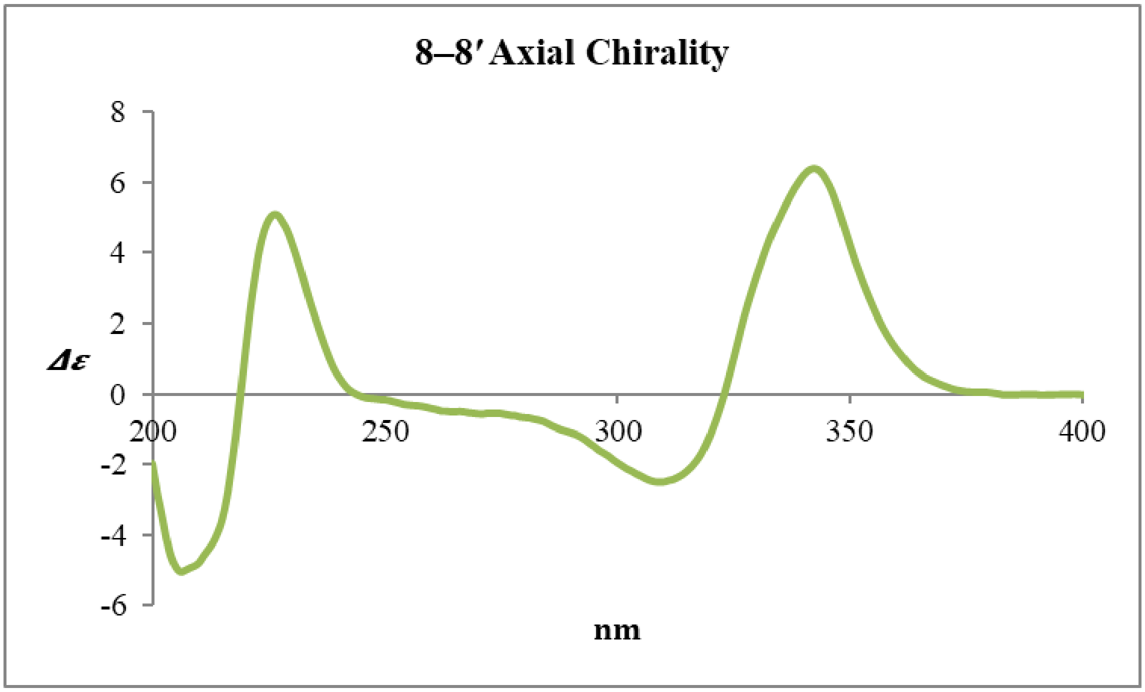

Figure S13. ECD spectrum of compound **6.**

$[\alpha]_D^{20}$= 30.9° (c 0.2, MeOH)

UV (MeOH) λmax (log ε) 325 (3.59) nm

ECD (MeOH) λmax (*Δε*) 206 (-5.06), 226 (5.10), 310 (-2.51), 342 (6.41) nm

HRESIMS m/z 643.1085 $[M - H]^-$ (calcd for $C_{33}H_{23}O_{14}$, 643.1088)

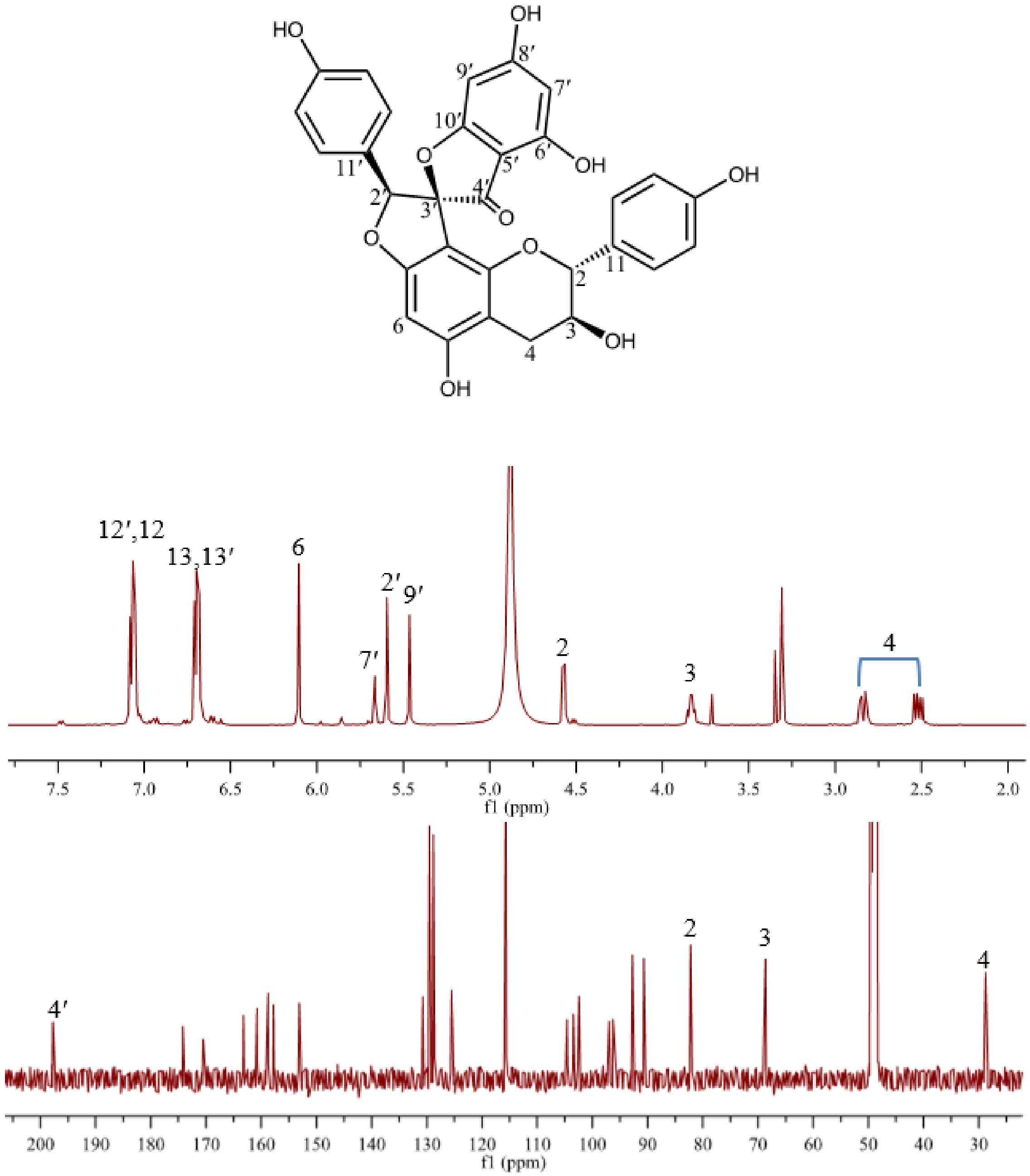

Figure S14. ^{1}H and ^{13}C NMR spectrum (MeOD-*d*4, 600/150 MHz) of compound **7.**

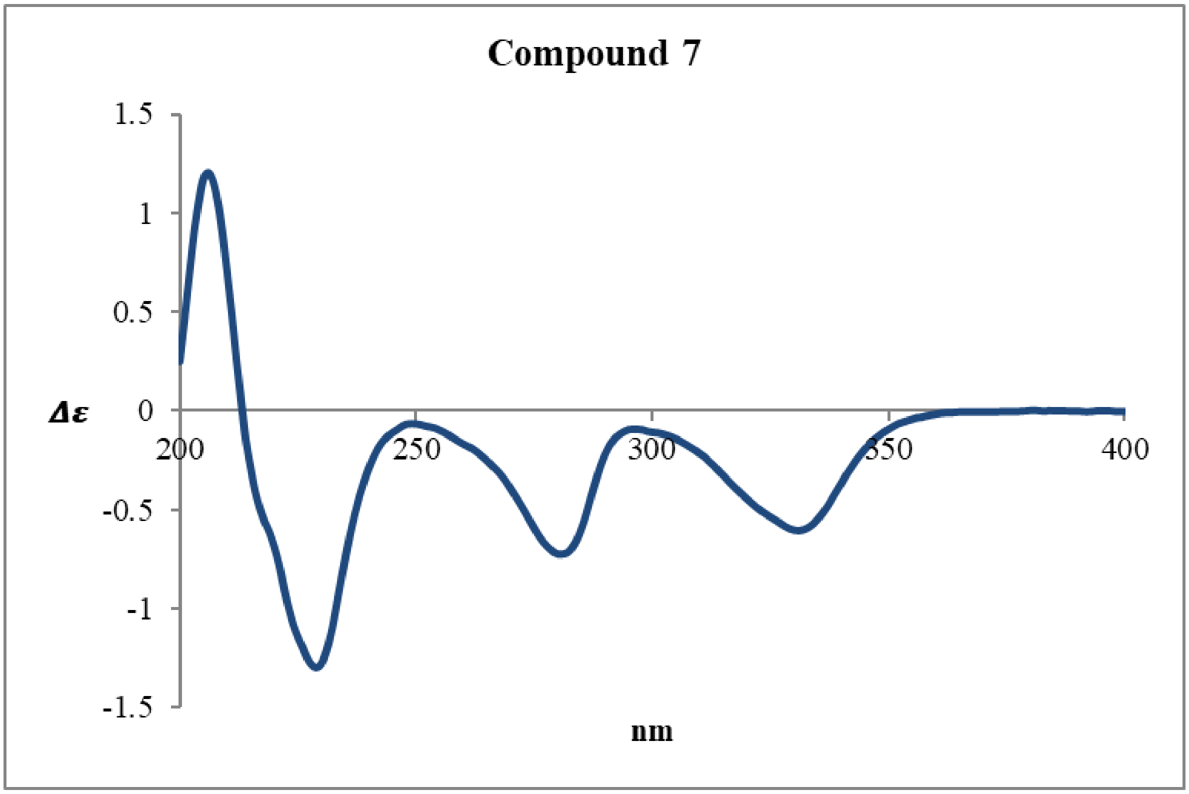

Figure S15. ECD spectrum of compound **7.**

$[\alpha]_D^{20}$= -265.6° (c 1.0, MeOH)

UV (MeOH) λmax (log ε) 216 (3.22), 286 (2.75) nm

ECD (MeOH) λmax (*Δε*) 204 (1.05), 229 (-1.30), 280 (-0.73), 331 (-0.61) nm

HRESIMS m/z 541.1159 $[M - H]^-$ (calcd for $C_{30}H_{21}O_{10}$, 541.1135)

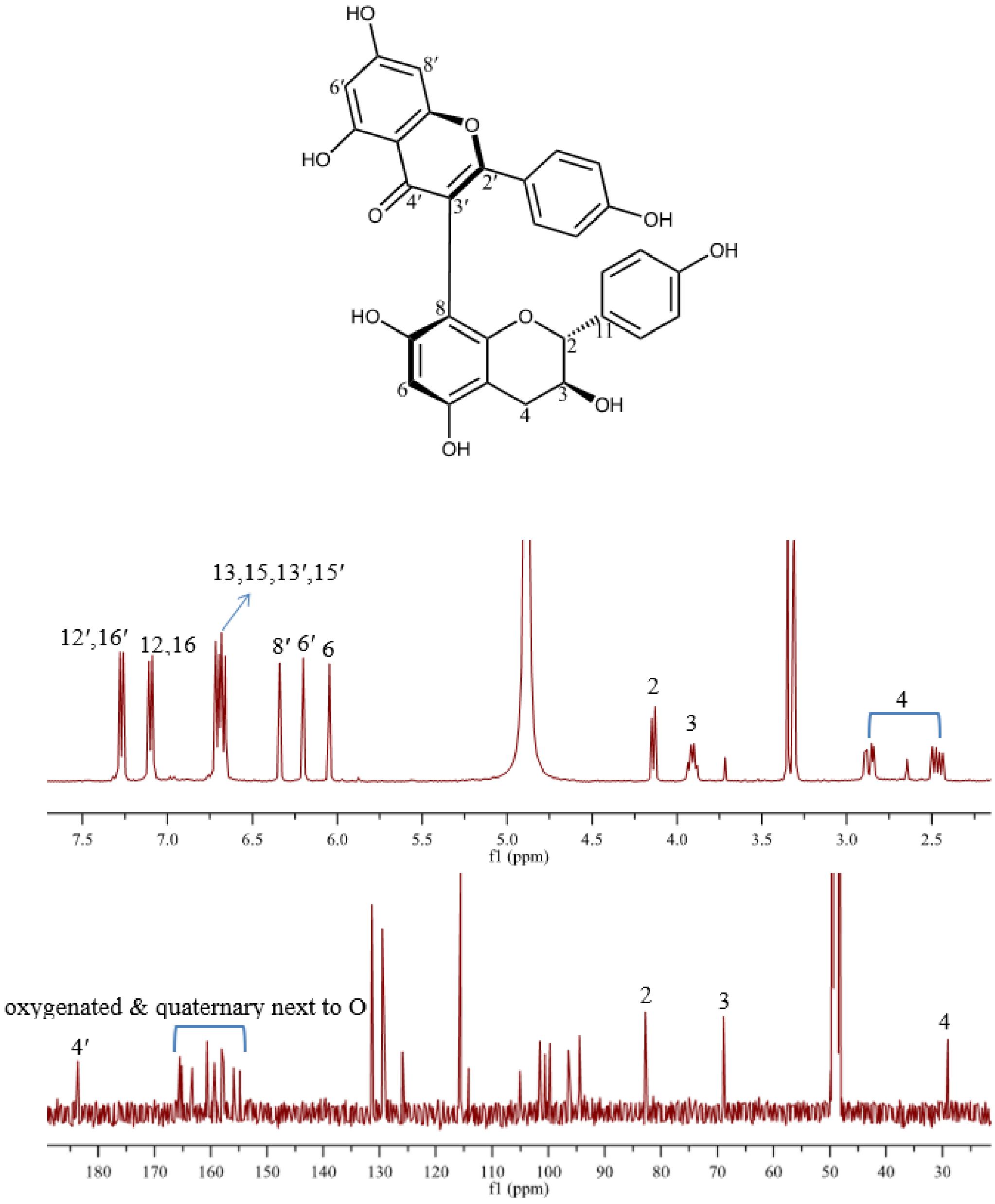

Figure S16. ^{1}H and ^{13}C NMR spectrum (MeOD-d_4, 400/100 MHz) of compound **8.**

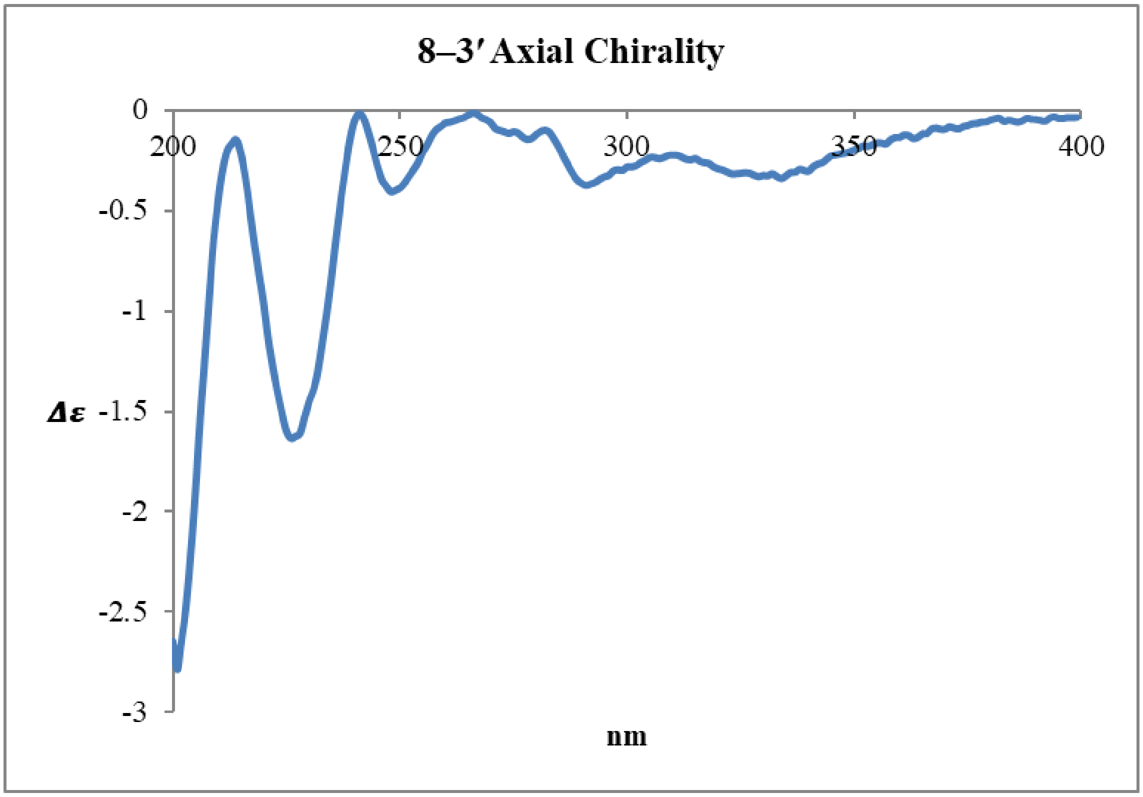

Figure S17. ECD spectrum of compound **8.**

$[\alpha]_D^{20}$= -31.2° (c 1.0, MeOH)

UV (MeOH) λmax (log ε) 207 (4.05), 266 (3.71), 331 (3.38) nm

ECD (MeOH) λmax (*Δε*) 226 (-1.63), 241 (-0.02) nm

HRESIMS m/z 541.1157 $[M - H]^-$ (calcd for $C_{30}H_{21}O_{10}$, 541.1135).

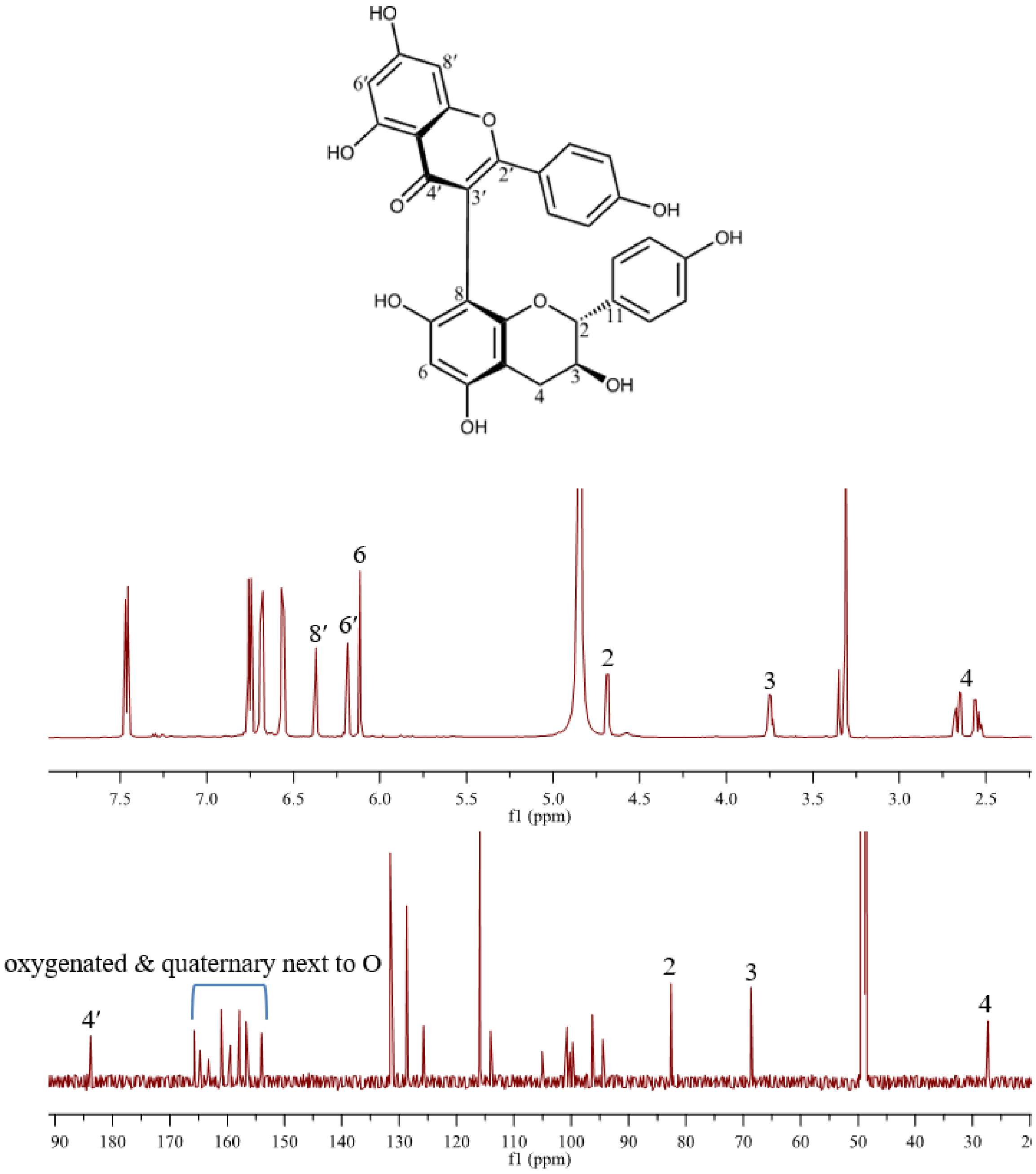

Figure S18. ^{1}H and ^{13}C NMR spectrum (DMSO-d_6, 600/150 MHz) of compound **9.**

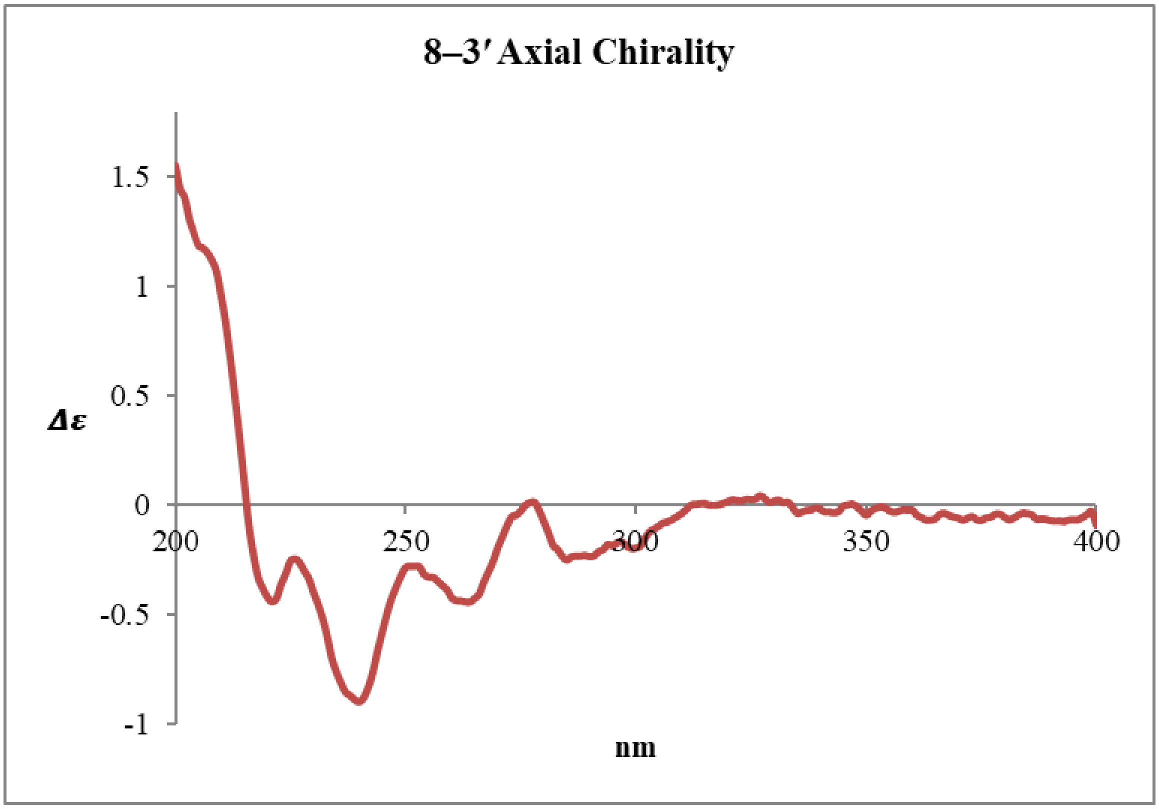

Figure S19. ECD spectrum of compound **9.**

$[\alpha]_D^{20}$= -13.6° (c 1.0, MeOH)

UV (MeOH) λmax (log ε) 210 (3.65), 266 (3.32), 333 (3.05) nm

ECD (MeOH) λmax (*Δε*) 226 (-0.49), 240 (-1.81) nm

HRESIMS m/z 541.1135 $[M - H]^-$ (calcd for $C_{30}H_{21}O_{10}$, 541.1135).

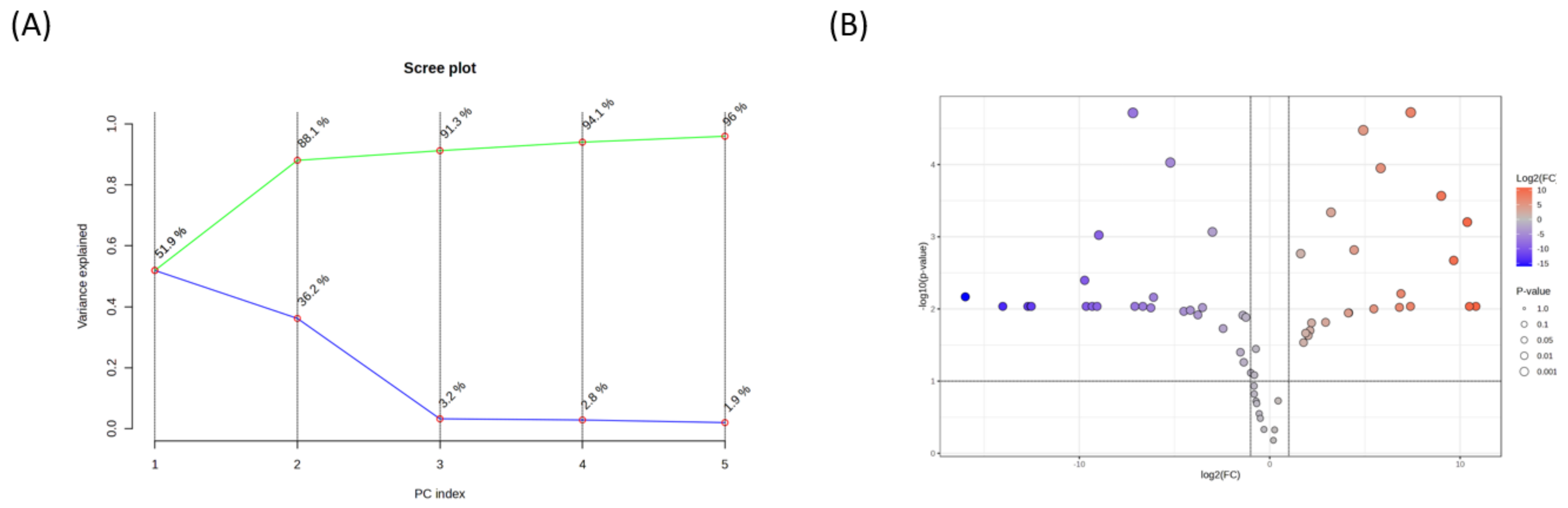

Fig. S20. Scree plot of PCA (A). Volcano plot of “Root” vs “leaf” (B).

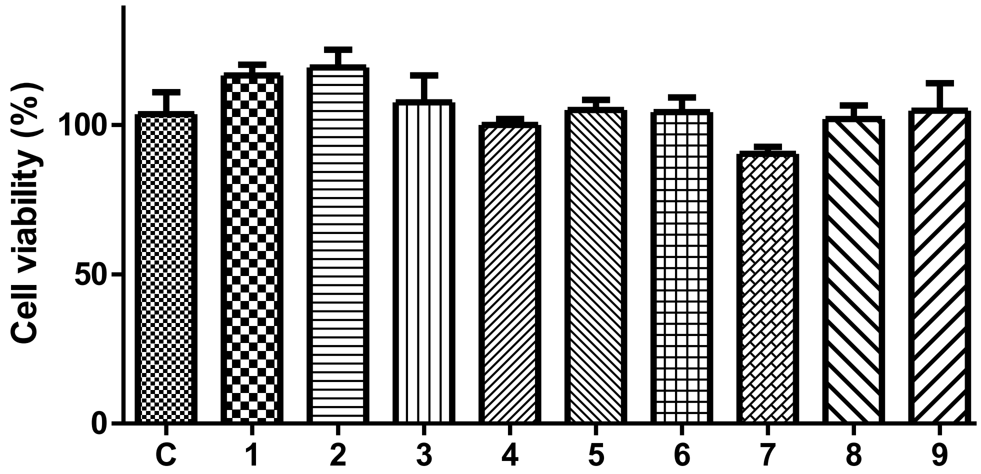

Figure S21. Effects of isolated compounds on cytotoxicity in 3T3-L1 adipocytes. The cells and test compounds at 20 μM were incubated for 24 h. The percentages of cell viability were evaluated using the MTT colorimetric assay.

Table S1. Structure and SMILES code of labeled compounds

no	name	structure	SMILES
**8**	CHEBI:182269	(2S,3R,4S,5S,6R)-2-[[(2S,3R)-3,5-dihydroxy-2-(4-hydroxyphenyl)-3,4-dihydro-2H-chromen-7-yl]oxy]-6-(hydroxymethyl)oxane-3,4,5-triol	C1[C@H]([C@@H](OC2=CC(=CC(=C21)O)O[C@H]3[C@@H]([C@H]([C@@H]([C@H](O3)CO)O)O)O)C4=CC=C(C=C4)O)O
**9**	NCGC00385785	(2R,3R,4S,5S,6R)-2-[[2-(3,4-dihydroxyphenyl)-5,7-dihydroxy-3,4-dihydro-2H-chromen-3-yl]oxy]-6-(hydroxymethyl)oxane-3,4,5-triol	C1C(C(OC2=CC(=CC(=C21)O)O)C3=CC(=C(C=C3)O)O)O[C@H]4[C@@H]([C@H]([C@@H]([C@H](O4)CO)O)O)O
**13**	NCGC00385785-01		O=C(O)C(N=C(O)NC1=NC=NC2=C1N=CN2C3OC(CO)C(O)C3O)C(O)C
**14**	NCGC00380504-01	7-[(2S,3R,4S,5S,6R)-6-[[(2R,3R,4R)-3,4-dihydroxy-4-(hydroxymethyl)oxolan-2-yl]oxymethyl]-3,4,5-trihydroxyoxan-2-yl]oxychromen-2-one	OC[C@@]1(O)CO[C@@H](OC[C@H]2O[C@@H](OC3=CC=C4C=CC(=O)OC4=C3)[C@H](O)[C@@H](O)[C@@H]2O)[C@@H]1O
**15**	NCGC00384579-01	7-[(2S,3R,4S,5S,6R)-3,4,5-trihydroxy-6-(hydroxymethyl)oxan-2-yl]oxychromen-2-one	O=C1OC=2C=C(OC3OC(CO)C(O)C(O)C3O)C=CC2C=C1
**17**	NCGC00380091	[2,6-dihydroxy-5-[3,4,5-trihydroxy-6-(hydroxymethyl)oxan-2-yl]oxycyclohex-3-en-1-yl] (E)-3-(3,4-dihydroxyphenyl)prop-2-enoate	C1=CC(C(C(C1O)OC(=O)/C=C/C2=CC(=C(C=C2)O)O)O)OC3C(C(C(C(O3)CO)O)O)O
**18**	NCGC00385380	3-[3,4-dihydroxy-6-(hydroxymethyl)-5-[3,4,5-trihydroxy-6-(hydroxymethyl)oxan-2-yl]oxyoxan-2-yl]oxy-2-(3,4-dihydroxyphenyl)-5,7-dihydroxychromen-4-one	C1=CC(=C(C=C1C2=C(C(=O)C3=C(C=C(C=C3O2)O)O)OC4C(C(C(C(O4)CO)OC5C(C(C(C(O5)CO)O)O)O)O)O)O)O
**20**	NCGC00381098	3-Hydroxy-1-(3-hydroxy-4-methoxyphenyl)-2-{4-[(1E)-3-hydroxy-1-propen-1-yl]-2-methoxyphenoxy}propyl beta-D-glucopyranoside	COC1=C(C=C(C=C1)C(C(CO)OC2=C(C=C(C=C2)/C=C/CO)OC)O[C@H]3[C@@H]([C@H]([C@@H]([C@H](O3)CO)O)O)O)O
**22**	NCGC00380686-01	5-hydroxy-7-[2-(2-hydroxy-5-oxo-2H-uran-3-yl)ethyl]-7,8-	CC1CCC23COC(=O)C2=CC(CC3C1(C)CCC4=CC(=O)OC4O)O

		dimethyl-5,6,6a,8,9,10-hexahydro-1H-benzo[d][2]benzouran-3-one	
**25**	NCGC00168890-02	6,7-dimethoxy-8-[(2S,3R,4S,5S,6R)-3,4,5-trihydroxy-6-(hydroxymethyl)oxan-2-yl]oxychromen-2-one	COC1=C(OC)C(O[C@@H]2O[C@H](CO)[C@@H](O)[C@H](O)[C@H]2O)=C3OC(=O)C=CC3=C1
**42**	NCGC00386047-01	3,5-dihydroxy-2-(4-hydroxyphenyl)-7-[(2R,3R,4S,5S,6R)-3,4,5-trihydroxy-6-(hydroxymethyl)oxan-2-yl]oxychromen-4-one	OC[C@H]1O[C@H](OC2=CC(O)=C3C(=O)C(O)=C(OC3=C2)C4=CC=C(O)C=C4)[C@H](O)[C@@H](O)[C@@H]1O
**49**	NCGC00380415-01	5,7-dihydroxy-2-(4-hydroxyphenyl)-3-[3,5,7-trihydroxy-2-(4-hydroxyphenyl)-3,4-dihydro-2H-chromen-8-yl]-2,3-dihydrochromen-4-one	C1C(C(OC2=C1C(=CC(=C2C3C(OC4=CC(=CC(=C4C3=O)O)O)C5=CC=C(C=C5)O)O)O)C6=CC=C(C=C6)O)O
**50**	NCGC00380474-01	5-hydroxy-2-(4-hydroxyphenyl)-7-[[(2R,3S)-3,5,8-trihydroxy-2-(4-hydroxyphenyl)-3,4-dihydro-2H-chromen-7-yl]oxy]chromen-4-one	C1[C@@H]([C@H](OC2=C1C(=CC(=C2O)OC3=CC(=C4C(=C3)OC(=CC4=O)C5=CC=C(C=C5)O)O)O)C6=CC=C(C=C6)O)O
**64**	NCGC00179820-03	2-(7-oxo-2,3-dihydrofuro[3,2-g]chromen-2-yl)propan-2-yl (Z)-2-methylbut-2-enoate	C/C=C(/C)\C(=O)OC(C)(C)C1CC2=C(O1)C=C3C(=C2)C=CC(=O)O3
